# This town ain't big enough for both of us…or is it? Spatial co-occurrence between exotic and native species in an urban reserve

**DOI:** 10.1371/journal.pone.0211050

**Published:** 2019-01-18

**Authors:** Gonzalo A. Ramírez-Cruz, Israel Solano-Zavaleta, Pedro E. Mendoza-Hernández, Marcela Méndez-Janovitz, Monserrat Suárez-Rodríguez, J. Jaime Zúñiga-Vega

**Affiliations:** 1 Posgrado en Ciencias Biológicas, Facultad de Ciencias, Universidad Nacional Autónoma de México, Ciudad Universitaria, Ciudad de México, Mexico; 2 Departamento de Ecología y Recursos Naturales, Facultad de Ciencias, Universidad Nacional Autónoma de México, Ciudad Universitaria, Ciudad de México, Mexico; 3 Posgrado en Ciencias Biológicas, Instituto de Ecología, Universidad Nacional Autónoma de México, Ciudad Universitaria, Ciudad de México, Mexico; Helmholtz Centre for Environmental Research - UFZ, GERMANY

## Abstract

Exotic species pose a threat to most ecosystems because of their potential to establish negative interactions with native biota. However, exotic species can also offer resources to native species, especially within highly modified environments such as urban ecosystems. We studied 17 exotic-native pairs of species with the potential to compete with one another, or in which one of the species could offer resources to the other, in an urban ecological reserve located within Mexico City. We used two-species occupancy models to analyze the potential association between the presence of the exotic species and the spatial distribution of the native species, as well as to assess if these species tend to avoid each other (negative spatial interaction) or to co-occur more often than expected under the hypothesis of independent occurrences (positive spatial interaction). Our results revealed few cases in which the exotic species influenced occupancy of the native species, and these spatial interactions were mainly positive, indicated by the fact that the occupancy of the native species was usually higher when the exotic species was also present. Seven of the eight observed non-independent patterns of co-occurrence were evident during the dry months of the year, when resources become scarce for most species. Our results also demonstrate that the observed patterns of species co-occurrence depend on the distance to the nearest urban structure and the amount of herb, shrub, and tree cover, indicating that these habitat features influence whether native species avoid or co-occur with exotic species. Our study represents an important contribution to the understanding of temporal dynamics in the co-occurrence between exotic and native species within urban ecological reserves.

## Introduction

Human activity has accelerated the introduction of non-native species in all types of ecosystems [1–4]. Multiple studies have gathered evidence of the negative effects of the presence of exotic species on native populations [5,6]. The introduction of a predator, parasite or competitor may have an evident impact on native animal populations [7–9]. Exotic plants in turn may create a variety of alterations to the local biotic composition, and can also become facilitators for the establishment of other non-native species [10,11]. However, there is also evidence for unexpected benefits brought by the introduction of exotic species [12]. For example, bird populations may benefit from the introduction of plant species that offer nesting sites and additional food resources [13]. Therefore, quantification of the associations between exotic species and native populations is necessary to implement management and conservation strategies [14–16].

Fragmentation of natural ecosystems by deforestation, agricultural activities, and urbanization is often accompanied by biological invasions [17–19]. Within fragmented areas, native species may be more susceptible to the effects of exotic species as a consequence of isolation, limited dispersal capabilities, and reduced resource availability [17,20,21]. Once established in fragmented habitats, exotic species may outcompete the native biota, causing local extinctions and threatening the functioning of ecosystems [3,22–24].

Urban areas contain novel assemblages of species that inhabit small patches of native vegetation within artificial environments [25–28]. Within cities, remnant woodlands usually function as reservoirs for species that are otherwise not found throughout the urban landscape [29,30]. These remnants of natural ecosystems contain species of flora and fauna that are representatives of the original biota [31]. However, human activities facilitate the introduction and establishment of non-native species into these isolated and highly-fragmented ecosystems [32]. Hence, these patches of native ecosystems within cities represent areas in which the impacts of exotic species might be exacerbated [25,33].

Mexico City is among the top five megacities of the world with respect to total human population [34]. Within this megacity, an ecological reserve (Reserva Ecológica del Pedregal de San Ángel, REPSA) was created in 1983, as an effort to preserve a unique xerophytic scrub that was formed after the eruption of the Xitle volcano almost 2 000 years ago [35–37]. Its 237 hectares are currently inhabited by 1 849 native species of plants and animals. Unfortunately, 317 exotic species have also been detected within its boundaries [38].

In this study, we asked whether the presence of exotic species is associated with the space use of native species in this ecological reserve immersed within a megacity. We focused on distinct pairs of exotic-native species that either are phylogenetically related, occupy similar ecological niches, or may provide resources to one another. We used two-species occupancy models [39–41] to estimate the probability that the occurrence of a given exotic species affects the occurrence of a native species (henceforth referred to as a spatial interaction), while accounting for their imperfect detection in the field [42].

In addition, we also asked if changes throughout the year in resource availability and climatic conditions might intensify or buffer the negative (or positive) spatial interactions between exotic and native species [5,43,44]. We predicted that the negative spatial interactions between exotic and native species that potentially compete for the same resources will be more intense during the most limiting season of the year and, therefore, native species would avoid areas where exotic species proliferate. In contrast, in those cases in which exotic species offer resources to native species, their positive spatial association would be stronger during the limiting season of the year. The possibility of seasonal dynamics in the intensity of the spatial interactions between exotic and native species remains highly unexplored. Therefore, we also asked whether the non-independent patterns of spatial co-occurrence between exotic and native species, estimated as the extent to which the presence of a given exotic species affects the presence of a native species, vary among three clearly distinct seasons: rainy, warm-dry, and cold-dry. For most species, wet conditions promote higher resource availability, whereas dry months entail limited resources [45,46].

## Material and methods

### Study area

**Fig 1:**
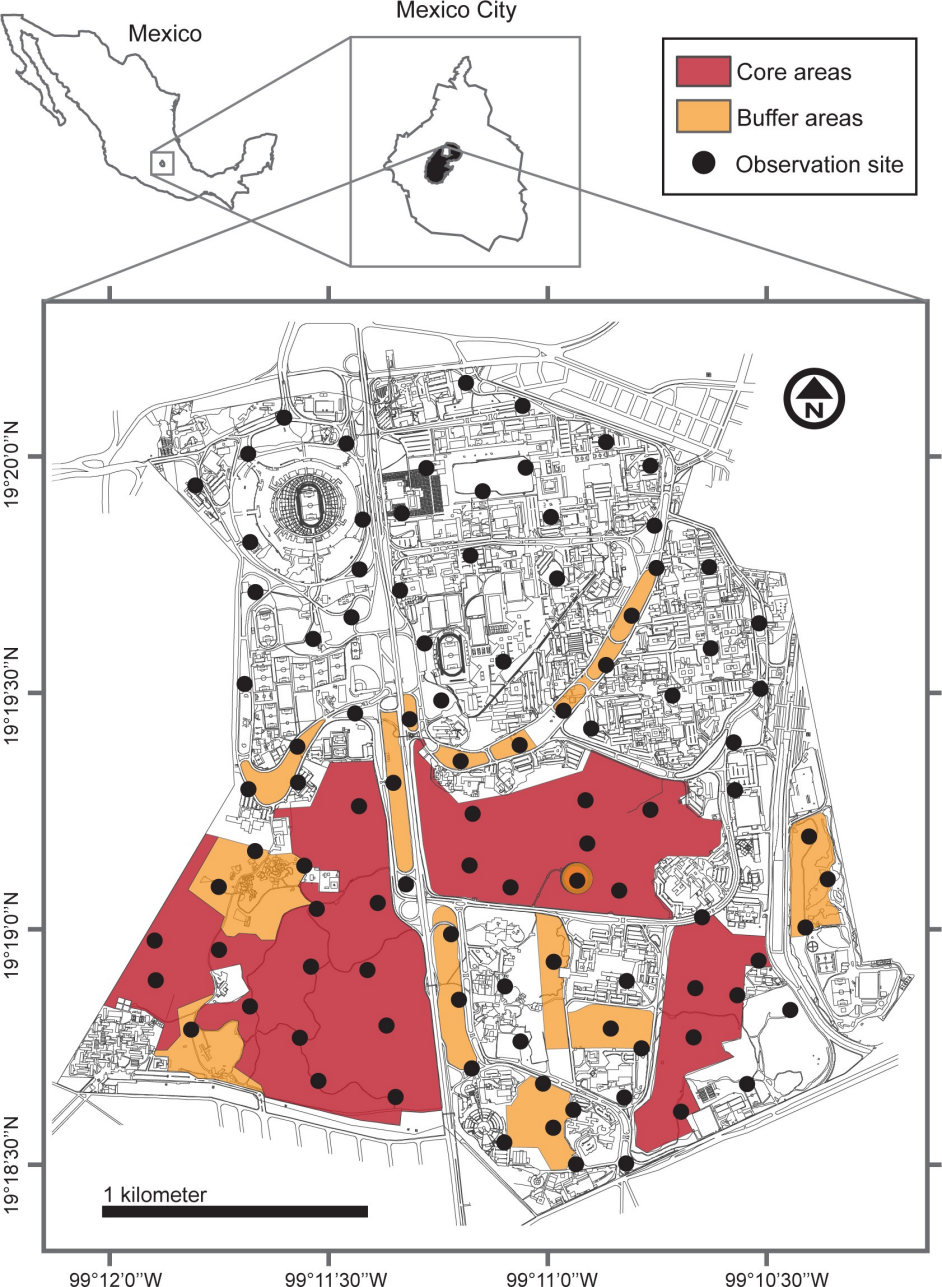
Location of the study area (Reserva Ecológica del Pedregal de San Ángel) within the main campus of Universidad Nacional Autónoma de México in Mexico City. We indicate the location of conservation, buffer, and urban areas. Circles depict observation sites. The black area within the insert of Mexico City shows the original extent of the native ecosystem.

The Reserva Ecológica del Pedregal de San Ángel (REPSA) is situated within the main campus of Universidad Nacional Autónoma de México (UNAM) in Mexico City (Fig 1). Climate in the area is temperate sub-humid with summer rains [47]. Mean annual temperature is 15.5°C. Total annual precipitation is on average 870 mm, with two distinct seasons: a rainy season from June to October (mean temperature = 16.7°C) and a dry season from November to May [48]. For this study we divided the dry season into two periods that clearly differ in their mean temperature: a cold-dry season from November to mid-February (mean temperature = 13.1°C) and a warm-dry season from mid-February to the end of May (mean temperature = 18.7°C) [49]. Local vegetation is a xerophytic scrub dominated by *Pittocaulon praecox* [37,50]. Since the mid-twentieth century, this native ecosystem has been fragmented due to rapid urban growth [51]. The reserve has a total extension of 237 ha, and is divided into three core areas (171 ha), and 13 buffer areas (66 ha) [52]. These conservation areas are surrounded by urban areas with multiple streets, avenues, artificial gardens, and buildings.

### Field methods

We established 100 observation sites distributed all over the core and buffer areas, as well as throughout the surrounding urban areas (Fig 1). The observation sites were randomly selected keeping a minimum distance between them of 150 m. We visited each site at five to seven occasions during May 2015, September 2015, January 2016, May 2016, September 2016, and January 2017. May represents the warm-dry season, September represents the rainy season, and January represents the cold-dry season. Therefore, our sampling scheme spanned two replicates of each season. Observations were carried out by a group of trained observers within a 20-m radius around the central point of each observation site. We detected species of flora and fauna within 15 minutes by using binoculars and through direct observation. At each site, we also measured the following habitat characteristics that we considered as environmental covariates in our occupancy analyses: percent cover of the herb (<0.5 m in height), shrub (0.5–3 m in height), and tree (>3 m in height) layers, average soil depth, percentage of litter, percentage of the substrate that consists of exposed volcanic rock, and the number of tree and shrub species. We also recorded whether the site is subjected to gardening activities, and measured the distance from the center of the site to the nearest building, garden, road, or human-made structure using Google Earth [53]. All data are freely available at Open Science Framework: https://osf.io/r5ket/.

### Study species

We selected several pairs of species formed by an exotic species and a species native to the reserve whose habits and characteristics make them prone to develop a spatial interaction (the predicted directions of these spatial interactions and the potentially underlying ecological interactions are summarized in Table 1). We based our selection of species on two criteria: (1) the species must have enough observations in the field to secure model convergence, and (2) must be easily identifiable to avoid false positives in our detection histories.

**Table 1:** Pairs of species for which we expected a non-independent pattern of co-occurrence.

Type of pair	Exotic species	Native species	Predicted spatial interaction	Potential ecological interaction
Bird-bird	House sparrow (*Passer domesticus*)	House finch (*Haemorhous mexicanus*)	Negative	Competition

		Inca dove (*Columbina inca*)	Negative	Competition
	
		American robin (*Turdus migratorius*)	Negative	Competition
	
		Bewick's wren (*Thryomanes bewickii*)	Negative	Competition
	
Mammal-mammal	Mexican red-bellied squirrel (*Sciurus aureogaster*)	Rock squirrel (*Otospermophilus variegatus*)	Negative	Competition

Grass-grass	Kikuyu grass (*Pennisetum clandestinum*)	Muhly grass (*Muhlenbergia robusta*)	Negative	Competition

	Rose natal grass (*Melinis repens*)		Negative	Competition
	
Tree-tree	Peruvian pepper (*Schinus molle*)	Tepozan tree (*Buddleja cordata*)	Negative	Competition

Tree-bird	Peruvian pepper (*Schinus molle*)	House finch (*Haemorhous mexicanus*)	Positive	Facilitation

		Grey silky-flycatcher (*Ptiliogonys cinereus*)	Positive	Facilitation
	
		Bushtit (*Psaltriparus minimus*)	Positive	Facilitation
	
	River red gum (*Eucalyptus camaldulensis*)	House finch (*Haemorhous mexicanus*)	Positive	Facilitation

		Grey silky-flycatcher (*Ptiliogonys cinereus*)	Positive	Facilitation
	
		Bushtit (*Psaltriparus minimus*)	Positive	Facilitation
	
	Tropical ash (*Fraxinus uhdei*)	House finch (*Haemorhous mexicanus*)	Positive	Facilitation

		Grey silky-flycatcher (*Ptiliogonys cinereus*)	Positive	Facilitation
	
		Bushtit (*Psaltriparus minimus*)	Positive	Facilitation
	

Each pair was conformed by an exotic and a native species that may co-occur more (positive spatial interaction) or less (negative spatial interaction) than expected under the hypothesis of independent occurrences.

#### Potential spatial interactions of the house sparrow with native birds

The house sparrow (*Passer domesticus*) is a bird native to Europe and North Africa. Its generalist diet, colonial nesting, aggressiveness towards other species, and affinity with human-altered landscapes have made it a successful invasive species throughout North America since its introduction in 1850 [54]. Previous studies have shown that avian communities in areas invaded by *P*. *domesticus* exhibit lower species richness compared to non-invaded areas [55]. We selected four native birds that according to their natural history and our own observations might co-occur in the same areas as *P*. *domesticus* (and thus might engage in an ecological interaction with this exotic bird) because they also exhibit an affinity for urban and disturbed areas.

The house finch (*Haemorhous mexicanus*) is a common species in North America that feeds mainly on seeds, fruits, leaves, and flowers, and nests on trees, cacti, buildings, and streetlights [56]. Given their similar diet and habits, some studies have documented negative interactions between the house sparrow and the house finch [56,57]. The Inca dove (*Columbina inca*) is a small dove native to Mexico, northern Central America, and the southwest of the United States. The Inca dove is a common inhabitant of arid environments and urbanized areas, where it has been observed to act aggressively towards the house sparrow [58]. We also considered two additional bird species: the American robin (*Turdus migratorius*), a migratory species that has established a resident population within our study area and that feeds primarily on invertebrates and fruits [59], and the Bewick’s wren (*Thryomanes bewickii*), a resident species that lives in scrub and forest areas, feeding on larvae and adult arthropods and building its nests in cavities and shrubs [60]. The house sparrow is known to usurp *T*. *migratorius* nests and has been reported to attack and molest other wren species in North America [57].

#### Potential spatial interaction of the Mexican red-bellied squirrel with the native rock squirrel

The Mexican red-bellied squirrel (*Sciurus aureogaster*) is a tropical tree squirrel native to the southeastern and southwestern coastal plains of Mexico and southwestern Guatemala. Its original distribution did not include our study area until 1999, when this squirrel colonized several areas of central Mexico [61], possibly as a result of the use of exotic trees for reforestation [62]. Unlike the introduced population of this tree squirrel in Florida, the Mexican red-bellied squirrels of central Mexico commonly explore the ground in search for food [62,63]. This behavior potentially promotes some degree of diet overlap with the native rock squirrel (*Otospermophilus variegatus*), a saxicolous species that is distributed from southeastern United States to central Mexico (including our study area) and that feeds on seeds, grain, roots, cacti, and invertebrates [64].

#### Potential spatial interactions of exotic grasses with the native muhly grass

The rose natal grass (*Melinis repens*) and the kikuyu grass (*Pennisetum clandestinum*) are two African grass species whose presence is associated with the decline of native plant diversity in certain areas, especially of other graminoids, and have the potential to displace other native grass species in dry habitats [65,66]. We expected these two exotic grass species to negatively affect the presence of the native muhly grass (*Muhlenbergia robusta*), which is a perennial grass that usually occurs in association with native shrubs and serves as shelter for many arthropod species [67].

#### Potential spatial interaction of the Peruvian pepper with the native tepozan tree

The Peruvian pepper (*Schinus molle*) is an evergreen tree native to the arid mountain slopes of South America whose first record in Mexico comes from the mid-sixteenth century [68]. This tree has the potential to become invasive and outcompete the native flora [69]. Because of its ability to establish and grow in shallow and rocky soils, our aim was to evaluate if the presence of the Peruvian pepper negatively affects the occurrence of the native tepozan tree (*Buddleja cordata*), an evergreen tree species distributed throughout Mexico [70] that is one of the most common species growing on the volcanic substrate of the reserve.

#### Potential spatial interactions of exotic trees with native birds

Exotic trees have the potential to offer food resources as well as additional nesting and perching sites to birds that live in urbanized environments [15,71]. For these reasons, we included three species of non-native trees that might promote the presence of native birds within our study area. First, the Peruvian pepper (*Schinus molle*) (see above) blooms and produces fruits all throughout the year and its seeds are frequently dispersed by birds [68]. Second, the river red gum (*Eucalyptus camaldulensis*), an Australian tree that has been extensively used for reforestation purposes in Mexico and is known to serve as a habitat for many birds [71]. Third, the tropical ash (*Fraxinus udhei*), native to mountain forests of Honduras, Guatemala, and western Mexico [72]; that is now a common exotic species within the REPSA and the gardening areas surrounding it [73]. The tropical ash is a well-known invasive species [74], with the capacity to alter the three-dimensional structure of the vegetation [75].

We considered three native bird species that might be attracted by these exotic trees. First, the house finch, which is one of the most common native birds in our study area (see above). Second, the grey silky-flycatcher (*Ptiliogonys cinereus*), which is a frugivorous montane species distributed from northwestern and eastern Mexico to Guatemala [76] and whose preference for forest-like areas makes it a common species in modified areas within the reserve where introduced trees are abundant. Third, the bushtit (*Psaltriparus minimus*), a small social insectivorous bird that gleans insects from the foliage in forests and shrublands [77].

### Data analyses

To estimate occupancy (ψ) and detection (*p*) probabilities for our focal species, we used the parameterization proposed by Richmond et al. [41] for two-species occupancy models. Using the detection histories (i.e., data on when any particular species was seen or not seen at each observation site) we built a conditional model in which species A, in this case the exotic species, is assumed to be dominant over species B, in this case the native species. We implemented these models separately for each season (i.e., two-species single-season occupancy models) in program MARK [78]. Environmental covariates with biological relevance for each pair of species were considered to model the following parameters: detection probability of the exotic species (*p*^A^), detection probability of the native species (*p*^B^), probability of occupancy of the exotic species (ψ^A^), probability of occupancy of the native species given that the exotic species is also present (ψ^BA^), and probability of occupancy of the native species given that the exotic species is absent (ψ^Ba^). In the case of tree-bird pairs, we also considered the detection probability of the bird given that the tree was present (*r*^B^). This was because the presence of the tree might affect the detectability of birds when they stop and perch as opposed to areas where tall trees are absent and birds are mainly detected in mid-flight, among the shrubs, or on the ground. In all other cases, we considered that the probability of detecting a focal species (either native or exotic) was independent of the presence of the other species in the pair (i.e., we set *r* = *p*).

We must notice here that although plants are sessile organisms, in all cases their detection probability was < 1, even for trees. This was because during our visits to the observation sites adult trees were easily detected, but seedlings and saplings were harder to see when surrounded by other vegetation and, hence, were not always detected when present. Also, changes among seasons in the occupancy of tree species were due to removal of adult individuals and establishment or mortality of seedlings and saplings [79].

We began our analyses by constructing a null model in which all parameters were held constant (i.e., estimating only an intercept for each parameter). Then, we built linear models to test the effect of all the biologically meaningful covariates on *p*, *r* and ψ. For both animals and plants, we considered the following covariates: percentage of tree, shrub, and herb cover, presence or absence of gardening activities (such as watering, pruning, and grass mowing), percentage of litter, distance to the nearest human-made structure, as well as number of tree species, and number of tree + shrub species present in the area. We also tested for differences in detection and occupancy probabilities among the three types of areas of the reserve. In the case of the Bewick’s wren, the rock squirrel, and plants we also considered the percentage of the substrate that consisted of exposed volcanic rock. In addition, for all plants we considered average soil depth as a relevant covariate. We used the Akaike's information criterion adjusted for small sample sizes (AICc; [80]) to select the best models for each species during each season (S1 Table). We considered that models that differed in less than two units of the AICc (ΔAICc < 2) with respect to the best-fitting model (i.e., the model with smallest AICc) also had strong support in the data [80].

Finally, we compared an unconditional model in which we set ψ^BA^ = ψ^Ba^, thereby assuming that the probability of occupancy of the native species is not affected by the presence of the exotic species (i.e., estimating a single ψ^B^), against a conditional model that included both ψ^BA^ and ψ^Ba^. These unconditional and conditional models incorporated the covariates that had the greatest effect on both occupancy and detection probabilities of each pair of species according to the models that we selected previously (S1 Table). If the conditional model had stronger support than the unconditional model (i.e., if the unconditional model had a ΔAICc > 2 with respect to the conditional model), we calculated a species interaction factor (SIF) to know if there is a positive or negative relationship between the occupancy of both species. The SIF is a measure of how likely are the species to co-occur in comparison to what would be expected given that their individual occurrences are independent [40,41,81]. For conditional models it can be calculated as:

$$SIF=\frac{\psi^{A}\psi^{BA}}{\psi^{A}(\psi^{A}\psi^{BA}+(1-\psi^{A})\psi^{Ba})}$$

where the observed co-occurrence (numerator in the equation) is divided by the expected co-occurrence assuming independence between the occupancies of both species (denominator in the equation; [41]). A SIF = 1 indicates that the two species occur independently. A SIF < 1 indicates that the native species is less likely to co-occur with the exotic species than expected under a hypothesis of independent occupancies (i.e., avoidance), whereas a SIF > 1 indicates that the native species tends to co-occur more frequently with the exotic species than expected under independence hypothesis (i.e., aggregation). Given that the patterns of species co-occurrence may depend on particular values of the environmental covariates, we show, for those pairs of species in which we detected a spatial interaction, the difference between the occupancy of the native species when the exotic species is present versus the occupancy of the native species when the exotic species is absent for different values of the most influential environmental covariates (as in Farris et al. [42]).

## Results

Overall, exotic species were detected in a greater proportion of the observation sites and were relatively more abundant than native species, except for the house finch and the tepozan tree (S2 Table). The house finch was detected in a similar proportion of sites and had a relative abundance similar to that of the exotic house sparrow. The tepozan tree was more abundant than any of the exotic trees that we studied (S2 Table).

Our results revealed few spatial interactions between native and exotic species (i.e., a few cases in which the conditional model had stronger support than the unconditional one). From a total of 17 pairs of species analyzed, we found evidence for spatial interaction only in six of these pairs. From these, we only found a non-independent pattern of co-occurrence in eight sampling periods (Tables 2 and 3), which represent only 7.8% of our total cases. In all other cases, the unconditional model was the top model or had relatively strong support in the data (i.e., ΔAICc < 2 with respect to the conditional model), meaning that including separate estimates for the occupancy probability of the natives depending on whether the exotics were present or absent did not improve substantially the model fit (Table 2).

**Table 2:** Summary of model selection results for two-species occupancy models that tested the hypothesis that the presence of an exotic species (denoted as A) influences the occupancy of a native species (denoted as B).

Exotic species	Native species	Warm-dry 2015	ΔAICc	Rainy 2015	ΔAICc	Cold-dry 2016	ΔAICc	Warm-dry 2016	ΔAICc	Rainy 2016	ΔAICc	Cold-dry 2017	ΔAICc
House sparrow (*Passer domesticus*)	House finch (*Haemorhous mexicanus*)	ψ^Ba^ = ψ^BA^	0.00	ψ^Ba^ = ψ^BA^	0.00	ψ^Ba^ ≠ ψ^BA^	0.00	ψ^Ba^ = ψ^BA^	0.00	ψ^Ba^ = ψ^BA^	0.00	**ψ**^**Ba**^ **≠ ψ**^**BA**^	**0.00**
		ψ^Ba^ ≠ ψ^BA^	4.90	ψ^Ba^ ≠ ψ^BA^	5.69	ψ^Ba^ = ψ^BA^	0.74	ψ^Ba^ ≠ ψ^BA^	1.51	ψ^Ba^ ≠ ψ^BA^	1.81	**ψ**^**Ba**^ **= ψ**^**BA**^	**8.16**
House sparrow (*Passer domesticus*)	Inca dove (*Columbina inca*)	ψ^Ba^ = ψ^BA^	0.00	ψ^Ba^ = ψ^BA^	0.00	ψ^Ba^ = ψ^BA^	0.00	ψ^Ba^ = ψ^BA^	0.00	ψ^Ba^ ≠ ψ^BA^	0.00	ψ^Ba^ = ψ^BA^	0.00
		ψ^Ba^ ≠ ψ^BA^	1.66	ψ^Ba^ ≠ ψ^BA^	2.48	ψ^Ba^ ≠ ψ^BA^	4.65	ψ^Ba^ ≠ ψ^BA^	4.33	ψ^Ba^ = ψ^BA^	0.95	ψ^Ba^ ≠ ψ^BA^	1.59
House sparrow (*Passer domesticus*)	American robin (*Turdus migratorius*)	ψ^Ba^ = ψ^BA^	0.00	ψ^Ba^ = ψ^BA^	0.00	ψ^Ba^ = ψ^BA^	0.00	ψ^Ba^ = ψ^BA^	0.00	ψ^Ba^ = ψ^BA^	0.00	ψ^Ba^ = ψ^BA^	0.00
		ψ^Ba^ ≠ ψ^BA^	7.23	ψ^Ba^ ≠ ψ^BA^	1.06	ψ^Ba^ ≠ ψ^BA^	4.26	ψ^Ba^ ≠ ψ^BA^	2.90	ψ^Ba^ ≠ ψ^BA^	2.36	ψ^Ba^ ≠ ψ^BA^	1.74
House sparrow (*Passer domesticus*)	Bewick's wren (*Thryomanes bewickii*)	ψ^Ba^ = ψ^BA^	0.00	ψ^Ba^ = ψ^BA^	0.00	ψ^Ba^ = ψ^BA^	0.00	ψ^Ba^ = ψ^BA^	0.00	ψ^Ba^ = ψ^BA^	0.00	ψ^Ba^ = ψ^BA^	0.00
		ψ^Ba^ ≠ ψ^BA^	4.87	ψ^Ba^ ≠ ψ^BA^	3.28	ψ^Ba^ ≠ ψ^BA^	3.91	ψ^Ba^ ≠ ψ^BA^	4.03	ψ^Ba^ ≠ ψ^BA^	3.46	ψ^Ba^ ≠ ψ^BA^	2.05
Mexican red-bellied squirrel (*Sciurus aureogaster*)	Rock squirrel (*Otospermophilus variegatus*)	**ψ**^**Ba**^ **≠ ψ**^**BA**^	**0.00**	ψ^Ba^ = ψ^BA^	0.00	ψ^Ba^ = ψ^BA^	0.00	ψ^Ba^ = ψ^BA^	0.00	**ψ**^**Ba**^ **≠ ψ**^**BA**^	**0.00**	ψ^Ba^ ≠ ψ^BA^	0.00
		**ψ**^**Ba**^ **= ψ**^**BA**^	**6.90**	ψ^Ba^ ≠ ψ^BA^	4.43	ψ^Ba^ ≠ ψ^BA^	4.28	ψ^Ba^ ≠ ψ^BA^	4.37	**ψ**^**Ba**^ **= ψ**^**BA**^	**10.50**	ψ^Ba^ = ψ^BA^	1.07
Rose natal grass (*Melinis repens*)	Muhly grass (*Muhlenbergia robusta*)	ψ^Ba^ = ψ^BA^	0.00	ψ^Ba^ = ψ^BA^	0.00	ψ^Ba^ = ψ^BA^	0.00	**ψ**^**Ba**^ **≠ ψ**^**BA**^	**0.00**	ψ^Ba^ ≠ ψ^BA^	0.00	ψ^Ba^ = ψ^BA^	0.00
		ψ^Ba^ ≠ ψ^BA^	1.52	ψ^Ba^ ≠ ψ^BA^	2.26	ψ^Ba^ ≠ ψ^BA^	3.44	**ψ**^**Ba**^ **= ψ**^**BA**^	**4.48**	ψ^Ba^ = ψ^BA^	0.39	ψ^Ba^ ≠ ψ^BA^	0.41
Kikuyu grass (*Pennisetum clandestinum*)	Muhly grass (*Muhlenbergia robusta*)	ψ^Ba^ = ψ^BA^	0.00	ψ^Ba^ = ψ^BA^	0.00	ψ^Ba^ = ψ^BA^	0.00	ψ^Ba^ = ψ^BA^	0.00	ψ^Ba^ = ψ^BA^	0.00	ψ^Ba^ = ψ^BA^	0.00
		ψ^Ba^ ≠ ψ^BA^	4.10	ψ^Ba^ ≠ ψ^BA^	3.75	ψ^Ba^ ≠ ψ^BA^	3.44	ψ^Ba^ ≠ ψ^BA^	2.57	ψ^Ba^ ≠ ψ^BA^	1.69	ψ^Ba^ ≠ ψ^BA^	3.34
Peruvian pepper (*Schinus molle*)	Tepozan tree (*Buddleja cordata*)	ψ^Ba^ = ψ^BA^	0.00	ψ^Ba^ = ψ^BA^	0.00	ψ^Ba^ ≠ ψ^BA^	0.00	ψ^Ba^ ≠ ψ^BA^	0.00	ψ^Ba^ = ψ^BA^	0.00	ψ^Ba^ = ψ^BA^	0.00
		ψ^Ba^ ≠ ψ^BA^	3.25	ψ^Ba^ ≠ ψ^BA^	0.75	ψ^Ba^ = ψ^BA^	0.59	ψ^Ba^ = ψ^BA^	0.71	ψ^Ba^ ≠ ψ^BA^	2.00	ψ^Ba^ ≠ ψ^BA^	2.67
Peruvian pepper (*Schinus molle*)	House finch (*Haemorhous mexicanus*)	ψ^Ba^ = ψ^BA^	0.00	ψ^Ba^ ≠ ψ^BA^	0.00	ψ^Ba^ = ψ^BA^	0.00	ψ^Ba^ = ψ^BA^	0.00	ψ^Ba^ = ψ^BA^	0.00	ψ^Ba^ = ψ^BA^	0.00
		ψ^Ba^ ≠ ψ^BA^	3.70	ψ^Ba^ = ψ^BA^	0.99	ψ^Ba^ ≠ ψ^BA^	5.12	ψ^Ba^ ≠ ψ^BA^	2.38	ψ^Ba^ ≠ ψ^BA^	2.47	ψ^Ba^ ≠ ψ^BA^	3.95
Peruvian pepper (*Schinus molle*)	Grey silky-flycatcher (*Ptiliogonys cinereus*)	ψ^Ba^ = ψ^BA^	0.00	ψ^Ba^ = ψ^BA^	0.00	ψ^Ba^ = ψ^BA^	0.00	ψ^Ba^ = ψ^BA^	0.00	ψ^Ba^ = ψ^BA^	0.00	ψ^Ba^ = ψ^BA^	0.00
		ψ^Ba^ ≠ ψ^BA^	4.70	ψ^Ba^ ≠ ψ^BA^	4.40	ψ^Ba^ ≠ ψ^BA^	2.43	ψ^Ba^ ≠ ψ^BA^	3.60	ψ^Ba^ ≠ ψ^BA^	0.55	ψ^Ba^ ≠ ψ^BA^	1.87
Peruvian pepper (*Schinus molle*)	Bushtit (*Psaltriparus minimus*)	ψ^Ba^ = ψ^BA^	0.00	ψ^Ba^ = ψ^BA^	0.00	ψ^Ba^ = ψ^BA^	0.00	ψ^Ba^ = ψ^BA^	0.00	ψ^Ba^ = ψ^BA^	0.00	ψ^Ba^ = ψ^BA^	0.00
		ψ^Ba^ ≠ ψ^BA^	1.90	ψ^Ba^ ≠ ψ^BA^	3.80	ψ^Ba^ ≠ ψ^BA^	2.26	ψ^Ba^ ≠ ψ^BA^	3.63	ψ^Ba^ ≠ ψ^BA^	0.60	ψ^Ba^ ≠ ψ^BA^	4.86
River red gum (*Eucalyptus camaldulensis*)	House finch (*Haemorhous mexicanus*)	ψ^Ba^ = ψ^BA^	0.00	ψ^Ba^ = ψ^BA^	0.00	ψ^Ba^ ≠ ψ^BA^	0.00	ψ^Ba^ = ψ^BA^	0.00	ψ^Ba^ = ψ^BA^	0.00	ψ^Ba^ = ψ^BA^	0.00
		ψ^Ba^ ≠ ψ^BA^	1.90	ψ^Ba^ ≠ ψ^BA^	4.77	ψ^Ba^ = ψ^BA^	0.83	ψ^Ba^ ≠ ψ^BA^	1.97	ψ^Ba^ ≠ ψ^BA^	2.04	ψ^Ba^ ≠ ψ^BA^	6.27
River red gum (*Eucalyptus camaldulensis*)	Grey silky-flycatcher (*Ptiliogonys cinereus*)	**ψ**^**Ba**^ **≠ ψ**^**BA**^	**0.00**	ψ^Ba^ = ψ^BA^	0.00	**ψ**^**Ba**^ **≠ ψ**^**BA**^	**0.00**	ψ^Ba^ = ψ^BA^	0.00	ψ^Ba^ = ψ^BA^	0.00	ψ^Ba^ = ψ^BA^	0.00
		**ψ**^**Ba**^ **= ψ**^**BA**^	**3.83**	ψ^Ba^ ≠ ψ^BA^	2.93	**ψ**^**Ba**^ **= ψ**^**BA**^	**2.02**	ψ^Ba^ ≠ ψ^BA^	5.18	ψ^Ba^ ≠ ψ^BA^	3.88	ψ^Ba^ ≠ ψ^BA^	5.13
River red gum (*Eucalyptus camaldulensis*)	Bushtit (*Psaltriparus minimus*)	ψ^Ba^ = ψ^BA^	0.00	ψ^Ba^ = ψ^BA^	0.00	ψ^Ba^ = ψ^BA^	0.00	ψ^Ba^ = ψ^BA^	0.00	ψ^Ba^ = ψ^BA^	0.00	ψ^Ba^ = ψ^BA^	0.00
		ψ^Ba^ ≠ ψ^BA^	4.07	ψ^Ba^ ≠ ψ^BA^	4.47	ψ^Ba^ ≠ ψ^BA^	2.35	ψ^Ba^ ≠ ψ^BA^	4.57	ψ^Ba^ ≠ ψ^BA^	4.07	ψ^Ba^ ≠ ψ^BA^	4.50
Tropical ash (*Fraxinus uhdei*)	House finch (*Haemorhous mexicanus*)	ψ^Ba^ = ψ^BA^	0.00	ψ^Ba^ = ψ^BA^	0.00	**ψ**^**Ba**^ **≠ ψ**^**BA**^	**0.00**	ψ^Ba^ ≠ ψ^BA^	0.00	ψ^Ba^ ≠ ψ^BA^	0.00	ψ^Ba^ = ψ^BA^	0.00
		ψ^Ba^ ≠ ψ^BA^	4.14	ψ^Ba^ ≠ ψ^BA^	6.63	**ψ**^**Ba**^ **= ψ**^**BA**^	**7.62**	ψ^Ba^ = ψ^BA^	1.09	ψ^Ba^ = ψ^BA^	1.06	ψ^Ba^ ≠ ψ^BA^	7.43
Tropical ash (*Fraxinus uhdei*)	Grey silky-flycatcher (*Ptiliogonys cinereus*)	ψ^Ba^ = ψ^BA^	0.00	ψ^Ba^ = ψ^BA^	0.00	ψ^Ba^ = ψ^BA^	0.00	ψ^Ba^ = ψ^BA^	0.00	ψ^Ba^ = ψ^BA^	0.00	**ψ**^**Ba**^ **≠ ψ**^**BA**^	**0.00**
		ψ^Ba^ ≠ ψ^BA^	4.51	ψ^Ba^ ≠ ψ^BA^	4.86	ψ^Ba^ ≠ ψ^BA^	1.61	ψ^Ba^ ≠ ψ^BA^	5.04	ψ^Ba^ ≠ ψ^BA^	2.99	**ψ**^**Ba**^ **= ψ**^**BA**^	**5.71**
Tropical ash (*Fraxinus uhdei*)	Bushtit (*Psaltriparus minimus*)	ψ^Ba^ = ψ^BA^	0.00	ψ^Ba^ = ψ^BA^	0.00	ψ^Ba^ = ψ^BA^	0.00	ψ^Ba^ ≠ ψ^BA^	0.00	ψ^Ba^ = ψ^BA^	0.00	ψ^Ba^ = ψ^BA^	0.00
		ψ^Ba^ ≠ ψ^BA^	3.91	ψ^Ba^ ≠ ψ^BA^	4.73	ψ^Ba^ ≠ ψ^BA^	1.86	ψ^Ba^ = ψ^BA^	0.50	ψ^Ba^ ≠ ψ^BA^	4.57	ψ^Ba^ ≠ ψ^BA^	3.90

For each pair of exotic-native species and for each season we used the Akaike’s information criterion adjusted for small sample sizes (AICc) to compare a model in which the occupancy of the native species (ψ^B^) depends on the presence (ψ^BA^) or absence (ψ^Ba^) of the exotic species (conditional model, denoted as ψ^Ba^ ≠ ψ^BA^) against a model in which the occupancy of the native species is independent of the presence of the exotic species (unconditional model, denoted as ψ^Ba^ = ψ^BA^). Models highlighted in bold type correspond to cases in which the conditional model had stronger support than the unconditional model (ΔAICc > 2).

**Table 3:** Species interaction factors (SIF) for pairs of exotic and native species during the seasons in which we detected a non-independent pattern of co-occurrence.

Pair of species	Season and year	Species interaction factor	95% Confidence interval
House sparrow (*Passer domesticus*)*–*house finch (*Haemorhous mexicanus*)	Cold-dry 2017	1.49	1.08–1.90
Mexican red-bellied squirrel (*Sciurus aureogaster*)*–*rock squirrel (*Otospermophilus variegatus*)	Warm-dry 2015	1.85	1.21–2.50
	Rainy 2016	2.31	1.32–3.29
Rose natal grass (*Melinis repens*)*–*muhly grass (*Muhlenbergia robusta*)	Warm-dry 2016	1.20	0.99–1.41
River red gum (*Eucalyptus camaldulensis*)*–*grey silky-flycatcher (*Ptiliogonys cinereus*)	Warm-dry 2015	1.19	0.20–2.17
	Cold-dry 2016	1.52	1.02–2.03
Tropical ash (*Fraxinus uhdei*)*–*house finch (*Haemorhous mexicanus*)	Cold-dry 2016 core areas	0.53	0.23–0.83
	Cold-dry 2016 buffer areas	0.61	0.31–0.91
	Cold-dry 2016 urban areas	0.79	0.57–1.01
Tropical ash (*Fraxinus uhdei*)*–*grey silky-flycatcher (*Ptiliogonys cinereus*)	Cold-dry 2017	0.65	0.33–0.97

In the case of the tropical ash (*Fraxinus uhdei*) and the house finch (*Haemorhous mexicanus*) we found slight differences among the three types of areas of the reserve (see S3 Table).

### Positive spatial interactions

The conditional model for the house sparrow and the house finch had stronger support than the unconditional model during the cold-dry season of 2017 (Table 2). The estimated SIF for this season was statistically greater than 1 (Table 3). During this season, occupancy probability of the house finch was higher where the house sparrow was also present than in sites where it was absent (ψ^BA^ = 0.88 ± 0.06 S.E. and ψ^Ba^ = 0.49 ± 0.09 S.E.). Also during this season, occupancy probability of the house sparrow was negatively affected by the distance to urban structures (β = -0.09, 95% C.I. = -0.15 –-0.04; S3 Table).

**Fig 2:**
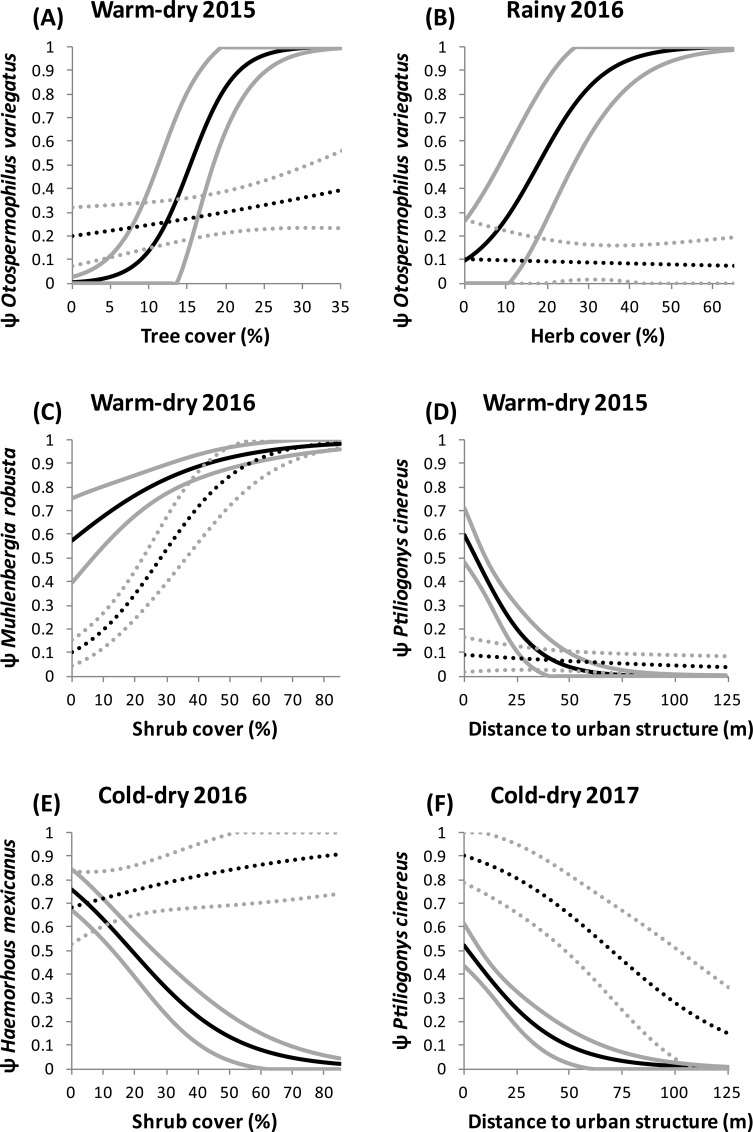
Effect of environmental covariates on the occupancy probability of native species in the presence (solid lines) and absence (dotted lines) of exotic species. Corresponding exotic species are: (A) and (B) Mexican red-bellied squirrel (*Sciurus aureogaster*), (C) rose natal grass (*Melinis repens*), (D) river red gum (*Eucalyptus camaldulensis*), (E) and (F) tropical ash (*Fraxinus uhdei*). Black and grey lines represent mean ± one standard error, respectively.

The conditional model for the Mexican red-bellied squirrel and the rock squirrel had stronger support than the unconditional model during two seasons: warm-dry of 2015 and rainy of 2016 (Table 2). Both estimated SIF values were statistically greater than 1 (Table 3). During the warm-dry season of 2015, the effect of tree cover on the occupancy of the native squirrel was higher when the exotic one was present (Fig 2A). Occupancy of the Mexican red-bellied squirrel was also positively affected by tree cover (β = 0.06, 95% C.I. = 0.02–0.1; S3 Table). During the rainy season of 2016, occupancy of the native squirrel was positively affected by herb cover where the exotic one was also present, compared to sites where it was absent (Fig 2B). During this last season, occupancy of the Mexican red-bellied squirrel was higher near urban structures (β = -0.03, 95% C.I. = -0.05 –-0.003; S3 Table).

Regarding the rose natal grass and the muhly grass, the conditional model had stronger support than the unconditional model only during the warm-dry season of 2016 (Table 2). The estimated SIF was greater than 1 and its confidence interval barely included 1 (Table 3). Shrub cover positively affected the occupancy probability of the muhly grass during this season, and was higher when the rose natal grass was also present (Fig 2C). Occupancy probability of the rose natal grass was also positively affected by shrub cover (β = 0.06, 95% C.I. = 0.03–0.08; S3 Table).

We found evidence of spatial interaction between the river red gum and the grey silky-flycatcher during the warm-dry season of 2015 and the cold-dry season of 2016. In both cases, the conditional model had stronger support than the unconditional model (ΔAICc > 2; Table 2). The estimated SIF for the latter season was statistically greater than 1, but the SIF for the former season was poorly estimated with a confidence interval that clearly included 1 (Table 3). During the warm-dry season of 2015, the negative effect of distance to urban structures on the occupancy of the grey silky-flycatcher was stronger where the river red gum was present than in sites where it was absent (Fig 2D). During the cold-dry season of 2016, occupancy probability of this bird was higher in sites where the river red gum was present compared to sites where this exotic tree was absent (ψ^BA^ = 0.34 ± 0.09 S.E. and ψ^Ba^ = 0.14 ± 0.05 S.E.). During both seasons, occupancy of the river red gum was negatively affected by the distance to urban structures (β = -0.03, 95% C.I. = -0.047 –-0.01, and β = -0.04, 95% C.I. = -0.06 –-0.02, respectively; S3 Table).

### Negative spatial interactions

We detected negative associations between the presence of the tropical ash and the occupancy probabilities of two species of native birds. The conditional model with the house finch had stronger support than the unconditional model during the cold-dry season of 2016 (Table 2). The estimated SIF varied between types of areas: those for core and buffer areas were statistically lower than 1, whereas the confidence interval for the SIF estimated for urban areas barely included 1 (Table 3). In sites where the tropical ash was present, occupancy probability of the house finch was negatively affected by shrub cover (Fig 2E). In contrast, in sites where this exotic tree was absent, occupancy of the house finch was substantially higher and had a positive relationship with shrub cover (Fig 2E). Occupancy of the tropical ash differed statistically between the core, buffer, and urban areas (ψ^A^ = 0.16, 95% C.I. = 0.05–0.40; ψ^A^ = 0.39, 95% C.I. = 0.19–0.64; ψ^A^ = 0.74, 95% C.I. = 0.59–0.85, respectively; S3 Table).

The conditional model for the tropical ash and the grey silky-flycatcher had stronger support than the unconditional model during the cold-dry season of 2017 (Table 2). The estimated SIF was statistically lower than 1 (Table 3). Occupancy probability of this bird was highest in urban areas and decreased as the distance to the nearest urban structure increased. In addition, its occupancy was lower in sites where the tropical ash was also present than in sites where this exotic tree was absent (Fig 2F). Occupancy probability of the tropical ash during this season was positively affected by gardening activities (β = 1.41, 95% C.I. = 0.21–2.61; S3 Table).

## Discussion

The evidence of associations between the presence of exotic species and the occupancy of native species in our study area is relatively small. In addition, the few non-independent patterns of species co-occurrence that we detected were evident only in one or two of the six sampling seasons. In other words, the spatial interactions between exotic and native species, as well as the environmental covariates associated with the observed non-independent co-occurrence patterns, were not consistent across years and seasons. This suggests that the underlying ecological interactions between exotic and native species are highly dynamic. We must also emphasize that in four of the six pairs of species in which we detected a spatial interaction, the association between presence of the exotic and occupancy of the native was positive (see Table 3).

Several authors have demonstrated how harmful exotic plants and animals are after becoming invasive in different ecosystems (e.g., [82–84]). However, some studies have documented cases in which the introduction of an exotic species has had little or no impact on native communities [85–87], indicating that exotic taxa do not always have negative interactions with native species that may drive changes in the composition or functioning of ecosystems. In fact, although less common within the literature on biological invasions, some studies have found that the presence of exotic species may provide certain benefits to native communities [88–90].

Our results deviate from most of the existing studies that have documented negative effects of exotic species on native populations [9,91,92]. Here, we found few spatial interactions and over a half of these were positive. We can explain these unexpected results in three ways. First, urban ecosystems are known to offer abundant resources (food and refuges) to both plants and animals, and this resource availability is usually constant all throughout the year [93,94]. Hence, negative interactions, such as competition, may be less intense in urban environments. In fact, the “credit-card hypothesis” poses that, given low predation and high food predictability in cities, urban species become less aggressive and more tolerant to the presence of other species [95–97].

Second, under certain ecological circumstances, mixed-species groups may have higher fitness than conspecific groups because they share both vigilance against predators and information on high-quality foraging patches [98,99]. This may be the case of the observed aggregations between the house finch and the house sparrow and between the Mexican red-bellied squirrel and the rock squirrel.

Third, most of the studies that have documented negative effects of exotic on native species are based on controlled experiments or have gathered evidence of competition for shared resources (e.g., [100–102]). Instead, we aimed to detect spatial patterns of co-occurrence, which is a novel approach to examine the consequences of ecological interactions on the distribution of species within particular areas. A handful of recent studies have also used two-species occupancy models in an attempt to demonstrate negative spatial interactions between exotic and native taxa (e.g., [103–105]). At least two of these studies also detected positive associations between the presence of exotic species and occupancy of native species (cats and dogs co-occur with some native carnivores in Madagascar; [42,106]). Therefore, future similar evaluations of spatial co-occurrences that take into account imperfect detectability of species in the field, may reveal that positive spatial interactions between exotic and native species are more common than previously thought.

We must emphasize here that two-species occupancy models provide robust statistical evidence of non-independent patterns of species co-occurrence [41], but these patterns do not represent direct evidence of the underlying ecological interactions, such as competition or facilitation [39]. However, the patterns that we detected here allowed us to propose hypotheses about the ecological processes that caused the observed spatial interactions. Formal tests of these hypotheses will require data on resource consumption by each pair of species and implementation of field experiments.

### Aggregation between exotic and native species

We found evidence of a positive spatial interaction between the house sparrow and the house finch during one of the cold-dry seasons. Given that these two birds are generalist granivores, the potential interaction between these two species has been widely studied with mixed results. For instance, Cooper et al. [107] found an inverse relationship between their abundances in northeastern United States, whereas McClure et al. [108] demonstrated that interspecific competition between these birds in southeastern United States is not strong enough to cause changes in their spatial distributions. Our results clearly suggest aggregation of the house sparrow and the house finch during the cold-dry season of 2017, with additional non-conclusive evidence of this same phenomenon during the previous cold-dry season of 2016 (see Table 2). We hypothesize that this aggregation pattern reflects that resources during the cold-dry seasons may be scarce, and thus the co-occurrence with each other may arise as a response to increased food availability provided by human activities in particular areas during winter.

We also found a positive spatial interaction between the two squirrels during two seasons. The Mexican red-bellied squirrel showed an evident affinity for forested areas near urban structures, which was expected for this exotic squirrel because the tree canopy is its primary habitat. Intriguingly, occupancy of the native rock squirrel, which lives among the rocks on the ground, was also related to tree and herb cover. Furthermore, occupancy of the rock squirrel increased substantially where the Mexican red-bellied squirrel was present and this co-occurrence was evident in sites where tree and herb cover are greater than approximately 20%. Likely, the Mexican red-bellied squirrel prefers areas in which particular tree and herb species are present that provide them with appropriate and abundant food resources. High food availability also attracts the presence of the native squirrel, which has a similar diet [62,64]. The observed aggregation between these two squirrels is noteworthy, because previous studies have reported the competitive exclusion of native squirrels by introduced ones [109,110]. However, we focused on two species of squirrels with different habitats (the Mexican red-bellied squirrel is predominantly arboreal and the rock squirrel is clearly saxicolous) and found that both seem to benefit from the same type of vegetation patches within our study area.

Two additional species that showed a positive spatial interaction were the exotic rose natal grass and the native muhly grass. These species were positively affected by shrub cover during one of the warm-dry seasons, when water becomes scarce and less available for all plants within the reserve. This indicates that the rose natal grass did not seem to compete for space or water with the muhly grass, but rather that they tend to co-occur in shrubland areas. In fact, in sites where shrub cover is less than approximately 35%, occupancy probability of the muhly grass was notably higher in the presence of the rose natal grass. Exotic grasses are known to modify the characteristics of the soil, thereby facilitating the growth of other plants (conspecifics, other exotic plants, and even native species), depending on the composition of the soil biota [111]. Hence, we suggest the hypothesis that in sites where shrub cover is scarce, the rose natal grass might facilitate the establishment of the native muhly grass through modification of the substrate. Alternatively, these two grasses may take advantage of soil patches with high nutrient levels and low density of shrubs, that allow them to coexist without evidence of competition [112]. In contrast, where shrub cover is abundant, the positive effect that it has on the occupancy probabilities of both species could reflect a nurse effect of native shrubs on these two grasses.

Finally, in two seasons we found a positive spatial interaction of the exotic river red gum with the grey silky-flycatcher, especially near human-made structures. This result is consistent with findings on how shrubland birds prefer exotic plant species as nesting sites [113]. Furthermore, this positive spatial interaction may be explained by the fact that the grey silky-flycatcher primarily inhabits montane forests, and is found in open areas only when scattered trees are present [114]. The river red gum is a tall tree that stands out among the shrubland vegetation and provides elevated roosting and perching sites for this native bird in the urban areas surrounding the reserve.

We must recognize that the positive co-occurrence patterns that we observed could also reflect neutral interactions, in which neither of the species involved receive a direct benefit [115]. Instead, these apparent aggregations may be either incidental or caused by shared habitat preferences. Even in the case that the observed positive co-occurrence patterns indeed arose from positive ecological interactions, such as mutualism or facilitation, our analyses ignored negative effects that our focal exotic species may have on other native taxa (e.g., native arthropods). Therefore, these positive spatial associations must not be interpreted as conclusive evidence of lack of negative impacts of these exotic species, and management plans should still focus on their monitoring and control.

### Spatial exclusion of native birds by an exotic tree

Negative spatial interactions occurred during both cold-dry seasons involving the exotic tropical ash and two species of native birds: the house finch and the grey silky-flycatcher. Previous studies have demonstrated that exotic plants can act as ecological traps for birds by offering them some benefits, but ultimately reducing nest survival by increasing the risk of nest predation [13,116,117]. However, we observed negative spatial interactions during two winters, when neither of these bird species was breeding. Therefore, we hypothesize that both species evaded areas where the tropical ash is present because other native trees and shrubs offer more appropriate resources during the limiting cold-dry season, such as has been observed in other bird species inhabiting shrubland areas that prefer native plants as microhabitats instead of exotic plants [118].

In fact, exotic plants may have direct negative effects on the diversity and abundance of native plant populations, which might indirectly affect the distribution of birds in particular areas [119,120]. Given that the dietary habits of the house finch and the grey silky-flycatcher are mainly based on fruits and seeds [56,114], we hypothesize that both birds tend to avoid sites where the tropical ash is present because this exotic tree modifies the surrounding vegetation with shrubs and herbs that do not provide the appropriate diversity and/or abundance of potential food items and, hence, these two native birds preferably forage on other species of trees and shrubs that can grow in sites where the tropical ash is absent. This tentative explanation is consistent with the fact that the occupancy of the house finch had a positive relationship with shrub cover in the absence of the tropical ash, whereas in contrast, the co-occurrence probability of the house finch and the tropical ash decreased drastically at high levels of shrub cover (see Fig 2E) because the composition of shrub species is likely different in areas where the tropical ash is present.

### Temporal dynamics in the observed non-independent patterns of co-occurrence

According to our results, the few spatial interactions that we observed between exotic and native species are temporarily dynamic. In other words, the non-independent co-occurrence patterns that we detected were evident only during one or two particular seasons, instead of being evident consistently across all seasons and years. Temporal changes in ecological interactions between species have been observed in other systems as well [121,122]. For instance, the interaction between a tree and a shrub in the semi-arid southwestern United States shifted from competition to facilitation depending on the abiotic conditions of the environment [121]. Likely, the temporal changes that we observed in co-occurrence patterns were driven by similar changes in other ecological factors that we did not measure and that can vary among seasons and years, such as food availability and abundance of predators and competitors.

Nonetheless, we initially predicted that both positive and negative spatial interactions would be more evident during the most limiting seasons (i.e., during the cold-dry and warm-dry seasons). We found some support for this hypothesis. From the eight cases in which we detected a spatial interaction, only one occurred during a rainy season. All other spatial interactions occurred during the cold-dry and the warm-dry seasons of the year, when the productivity of the native vegetation decreases considerably, which in turn reduces resource availability and intensifies interspecific interactions (e.g., [123]).

Finally, our results also indicate that habitat preferences of our focal species also vary among seasons and years. This may be partially explained by the fact that urban ecosystems are subject to severe and constant modifications due to human activity [28]. Previous studies have confirmed that some birds are able to shift their habitat preferences according to the available resources from one season or year to the next [124,125]. Furthermore, we have also demonstrated that the observed non-independent co-occurrence patterns between exotic and native species depend on specific habitat features. In particular, coexistence of our focal species in this urban reserve immersed within Mexico City is apparently influenced by the amount of herb, shrub, and tree cover as well as by the proximity to human-made structures. Our results confirm the observations of Haynes et al. [126] and Estevo et al. [127] that the characteristics of the environment may influence whether different species avoid or co-occur with each other.

## Supporting information

S1 Table Model selection results for single-species occupancy models that tested the effects of environmental covariates on occupancy (ψ) and detection (*p*) probabilities of seven exotic and nine native species during six sampling periods. For each species and for each season we show models with strong support (ΔAICc < 2).
(DOCX)

S2 Table Number of sites where the study species were detected and their relative abundances estimated as the average number of detected individuals across six sampling periods. To avoid the double counting of animal individuals in the field, counts were made by a single observer within the first five minutes of the observation period. *In the case of grasses, we could not count individuals.
(DOCX)

S3 Table **Complete model selection results for two-species occupancy models that tested the hypothesis that the presence of exotic species (denoted as A) influences the occupancy of native species (denoted as B).** For each pair of exotic-native species and for each season we used the Akaike’s information criterion adjusted for small sample sizes (AICc) to compare a model in which the occupancy (ψ) of the native species depends on the presence (ψ^BA^) or absence (ψ^Ba^) of the exotic species against a model in which the occupancy of the native species is independent of the presence of the exotic species (ψ^B^). The environmental covariates with strongest effect on the parameters are shown within parentheses.
(DOCX)
