# Supplementary material for: This town ain't big enough for both of us…or is it? Spatial co-occurrence between exotic and native species in an urban reserve
Source: PLoS One. 2019 Jan 18;14(1):e0211050. doi: 10.1371/journal.pone.0211050 (PMC6338412; doi:10.1371/journal.pone.0211050)
Supplement: S1 Table — For each species and for each season we show models with strong support (ΔAICc < 2). (DOCX) [file pone.0211050.s001.docx]

**S1 Table. Model selection results for single-species occupancy models that tested the effects of environmental covariates on occupancy (ψ) and detection (*p*) probabilities of seven exotic and nine native species during six seasons.**

| **Species and season** | **Model** | **AICc** | **ΔAICc** | ***w*** | ***k*** |
| --- | --- | --- | --- | --- | --- |
| House sparrow (*Passer domesticus*) |  |  |  |  |  |
| Warm-Dry 2015 | ψ (gardening activities) *p* (distance to urban structure) | 540.6609 | 0 | 0.6086 | 4 |
| Rainy 2015 | ψ (shrub cover) *p* (distance to urban structure) | 398.7865 | 0 | 0.77873 | 4 |
| Cold-Dry 2016 | ψ (shrub cover) *p* (gardening activities) | 571.2422 | 0 | 0.28468 | 4 |
|  | ψ (gardening activities) *p* (type of area) | 571.8726 | 0.6304 | 0.20771 | 4 |
| Warm-Dry 2016 | ψ (distance to urban structure) *p* (type of area) | 581.9584 | 0 | 0.29376 | 5 |
|  | ψ (litter) *p* (type of area) | 582.1105 | 0.1521 | 0.27225 | 5 |
|  | ψ (tree richness) *p* (type of area) | 583.0838 | 1.1254 | 0.16735 | 5 |
| Rainy 2016 | ψ (type of area) *p* (shrub cover) | 656.0047 | 0 | 0.69855 | 5 |
| Cold-Dry 2017 | ψ (distance to urban structure) *p* (shrub cover) | 513.3615 | 0 | 0.1916 | 4 |
|  | ψ (tree cover) *p* (type of area) | 514.0647 | 0.7032 | 0.13481 | 4 |
|  | ψ (intercept only) *p* (type of area) | 514.1454 | 0.7839 | 0.12947 | 3 |
|  | ψ (type of area) *p* (type of area) | 514.4651 | 1.1036 | 0.11035 | 4 |
| House finch (*Haemorhous mexicanus*) |  |  |  |  |  |
| Warm-Dry 2015 | ψ (shrub cover) *p* (type of area) | 644.735 | 0 | 0.7745 | 5 |
| Rainy 2015 | ψ (type of area) *p* (herb cover) | 537.5721 | 0 | 0.06823 | 5 |
|  | ψ (type of area) *p* (tree cover) | 537.7013 | 0.1292 | 0.06396 | 5 |
|  | ψ (type of area) *p* (distance to urban structure) | 537.702 | 0.1299 | 0.06394 | 5 |
|  | ψ (type of area) *p* (intercept only) | 538.9638 | 1.3917 | 0.03402 | 4 |
|  | ψ (type of area) *p* (gardening activities) | 539.2312 | 1.6591 | 0.02976 | 5 |
| Cold-Dry 2016 | ψ (shrub cover) *p* (shrub cover) | 553.309 | 0 | 0.11625 | 4 |
|  | ψ (intercept only) *p* (shrub cover) | 553.4068 | 0.0978 | 0.1107 | 3 |
|  | ψ (distance to urban structure) *p* (shrub cover) | 554.1442 | 0.8352 | 0.07657 | 4 |
|  | ψ (herb cover) *p* (shrub cover) | 554.3941 | 1.0851 | 0.06757 | 4 |
|  | ψ (tree richness) *p* (shrub cover) | 554.9157 | 1.6067 | 0.05206 | 4 |
|  | ψ (tree cover) *p* (shrub cover) | 555.0777 | 1.7687 | 0.04801 | 4 |
|  | ψ (tree and shrub richness) *p* (shrub cover) | 555.2301 | 1.9211 | 0.04449 | 4 |
| Warm-Dry 2016 | ψ (intercept only) *p* (shrub cover) | 710.7734 | 0 | 0.13272 | 3 |
|  | ψ (tree and shrub richness) *p* (shrub cover) | 711.9686 | 1.1952 | 0.07301 | 4 |
|  | ψ (tree richness) *p* (shrub cover) | 711.9713 | 1.1979 | 0.07291 | 4 |
|  | ψ (distance to urban structure) *p* (shrub cover) | 712.1934 | 1.42 | 0.06525 | 4 |
|  | ψ (gardening activities) *p* (shrub cover) | 712.4973 | 1.7239 | 0.05605 | 4 |
| Rainy 2016 | ψ (intercept only) *p* (distance to urban structure) | 945.7675 | 0 | 0.15614 | 3 |
|  | ψ (tree cover) *p* (distance to urban structure) | 945.854 | 0.0865 | 0.14953 | 4 |
|  | ψ (herb cover) *p* (distance to urban structure) | 946.2222 | 0.4547 | 0.12439 | 4 |
|  | ψ (shrub cover) *p* (distance to urban structure) | 946.5056 | 0.7381 | 0.10795 | 4 |
|  | ψ (tree and shrub richness) *p* (distance to urban structure) | 946.611 | 0.8435 | 0.10241 | 4 |
|  | ψ (distance to urban structure) *p* (distance to urban structure) | 947.4381 | 1.6706 | 0.06772 | 4 |
| Cold-Dry 2017 | ψ (type of area) *p* (distance to urban structure) | 678.9829 | 0 | 0.0981 | 5 |
|  | ψ (intercept only) *p* (distance to urban structure) | 679.518 | 0.5351 | 0.07507 | 3 |
|  | ψ (type of area) *p* (type of area) | 680.2906 | 1.3077 | 0.05101 | 6 |
|  | ψ (litter) *p* (distance to urban structure) | 680.3103 | 1.3274 | 0.05051 | 4 |
| Inca dove (*Columbina inca*) |  |  |  |  |  |
| Warm-Dry 2015 | ψ (shrub cover) *p* (distance to urban structure) | 373.3035 | 0 | 0.15951 | 4 |
|  | ψ (type of area) *p* (distance to urban structure) | 373.5522 | 0.2487 | 0.14085 | 4 |
|  | ψ (type of area) *p* (tree and shrub richness) | 373.7034 | 0.3999 | 0.1306 | 4 |
|  | ψ (type of area) *p* (intercept only) | 374.1538 | 0.8503 | 0.10426 | 3 |
|  | ψ (type of area) *p* (exposed rock) | 374.5013 | 1.1978 | 0.08764 | 4 |
|  | ψ (type of area) *p* (gardening activities) | 375.0969 | 1.7934 | 0.06506 | 4 |
| Rainy 2015 | ψ (gardening activities) *p* (shrub cover) | 322.3339 | 0 | 0.16292 | 4 |
|  | ψ (distance to urban structure) *p* (shrub cover) | 323.0385 | 0.7046 | 0.11454 | 4 |
|  | ψ (shrub cover) *p* (type of area) | 323.3626 | 1.0287 | 0.09741 | 5 |
|  | ψ (type of area) *p* (shrub cover) | 323.595 | 1.2611 | 0.08672 | 5 |
|  | ψ (shrub cover) *p* (shrub cover) | 323.6261 | 1.2922 | 0.08538 | 4 |
| Cold-Dry 2016 | ψ (distance to urban structure) *p* (tree and shrub richness) | 246.0263 | 0 | 0.14384 | 4 |
|  | ψ (shrub cover) *p* (tree and shrub richness) | 246.0944 | 0.0681 | 0.13903 | 4 |
|  | ψ (type of area) *p* (exposed rock) | 247.6584 | 1.6321 | 0.0636 | 4 |
|  | ψ (type of area) *p* (shrub cover) | 247.8007 | 1.7744 | 0.05923 | 4 |
| Warm-Dry 2016 | ψ (distance to urban structure) *p* (exposed rock) | 339.6485 | 0 | 0.30003 | 4 |
|  | ψ (gardening activities) *p* (exposed rock) | 341.0157 | 1.3672 | 0.15145 | 4 |
| Rainy 2016 | ψ (shrub cover) *p* (distance to urban structure) | 473.2226 | 0 | 0.86755 | 4 |
| Cold-Dry 2017 | ψ (shrub cover) *p* (tree and shrub richness) | 278.379 | 0 | 0.08197 | 4 |
|  | ψ (type of area) *p* (gardening activities) | 278.4005 | 0.0215 | 0.08109 | 4 |
|  | ψ (shrub cover) *p* (distance to urban structure) | 278.7685 | 0.3895 | 0.06746 | 4 |
|  | ψ (gardening activities) *p* (distance to urban structure) | 279.3248 | 0.9458 | 0.05108 | 4 |
|  | ψ (intercept only) *p* (gardening activities) | 279.3513 | 0.9723 | 0.05041 | 2 |
|  | ψ (gardening activities) *p* (intercept only) | 279.3513 | 0.9723 | 0.05041 | 2 |
|  | ψ (gardening activities) *p* (gardening activities) | 279.3513 | 0.9723 | 0.05041 | 2 |
|  | ψ (shrub cover) *p* (tree richness) | 279.4935 | 1.1145 | 0.04695 | 4 |
|  | ψ (distance to urban structure) *p* (gardening activities) | 279.6089 | 1.2299 | 0.04432 | 3 |
|  | ψ (tree cover) *p* (gardening activities) | 279.7796 | 1.4006 | 0.04069 | 3 |
|  | ψ (gardening activities) *p* (exposed rock) | 279.9707 | 1.5917 | 0.03698 | 3 |
|  | ψ (shrub cover) *p* (type of area) | 280.0924 | 1.7134 | 0.0348 | 4 |
|  | ψ (type of area) *p* (tree richness) | 280.2449 | 1.8659 | 0.03225 | 4 |
| American robin (*Turdus migratorius*) |  |  |  |  |  |
| Warm-Dry 2015 | ψ (type of area) *p* (herb cover) | 446.2039 | 0 | 0.97306 | 5 |
| Rainy 2015 | ψ (shrub cover) *p* (tree richness) | 167.0755 | 0 | 0.30349 | 4 |
| Cold-Dry 2016 | ψ (shrub cover) *p* (exposed rock) | 204.0751 | 0 | 0.16306 | 4 |
|  | ψ (shrub cover) *p* (tree richness) | 205.0983 | 1.0232 | 0.09776 | 4 |
| Warm-Dry 2016 | ψ (type of area) *p* (herb cover) | 505.3383 | 0 | 0.62222 | 5 |
|  | ψ (type of area) *p* (type of area) | 506.8996 | 1.5613 | 0.28504 | 5 |
| Rainy 2016 | ψ (tree and shrub richness) *p* (shrub cover) | 247.9808 | 0 | 0.05079 | 4 |
|  | ψ (intercept only) *p* (shrub cover) | 249.3147 | 1.3339 | 0.02607 | 3 |
|  | ψ (intercept only) *p* (distance to urban structure) | 249.6202 | 1.6394 | 0.02238 | 3 |
|  | ψ (shrub cover) *p* (shrub cover) | 249.7366 | 1.7558 | 0.02111 | 4 |
|  | ψ (tree and shrub richness) *p* (distance to urban structure) | 249.8809 | 1.9001 | 0.01964 | 4 |
| Cold-Dry 2017 | ψ (distance to urban structure) *p* (intercept only) | 296.9571 | 0 | 0.08141 | 3 |
|  | ψ (distance to urban structure) *p* (tree and shrub richness) | 297.4448 | 0.4877 | 0.06379 | 4 |
|  | ψ (distance to urban structure) *p* (exposed rock) | 297.8022 | 0.8451 | 0.05335 | 4 |
|  | ψ (distance to urban structure) *p* (tree cover) | 297.8529 | 0.8958 | 0.05202 | 4 |
|  | ψ (distance to urban structure) *p* (distance to urban structure) | 298.1496 | 1.1925 | 0.04484 | 4 |
|  | ψ (distance to urban structure) *p* (gardening activities) | 298.1623 | 1.2052 | 0.04456 | 4 |
|  | ψ (distance to urban structure) *p* (herb cover) | 298.4261 | 1.469 | 0.03905 | 4 |
|  | ψ (distance to urban structure) *p* (type of area) | 298.7935 | 1.8364 | 0.0325 | 5 |
|  | ψ (distance to urban structure) *p* (tree richness) | 298.9138 | 1.9567 | 0.0306 | 4 |
|  | ψ (shrub cover) *p* (intercept only) | 298.9446 | 1.9875 | 0.03014 | 3 |
| Bewick's wren (*Thryomanes bewickii*) |  |  |  |  |  |
| Warm-Dry 2015 | ψ (tree cover) *p* (distance to urban structure) | 276.7144 | 0 | 0.0627 | 4 |
|  | ψ (tree cover) *p* (type of area) | 277.2257 | 0.5113 | 0.04856 | 5 |
|  | ψ (type of area) *p* (type of area) | 277.7205 | 1.0061 | 0.03791 | 5 |
|  | ψ (tree cover) *p* (exposed rock) | 278.2132 | 1.4988 | 0.02964 | 4 |
|  | ψ (tree cover) *p* (intercept only) | 278.4775 | 1.7631 | 0.02597 | 3 |
|  | ψ (intercept only) *p* (type of area) | 278.5717 | 1.8573 | 0.02477 | 4 |
| Rainy 2015 | ψ (litter) *p* (tree richness) | 300.0815 | 0 | 0.11742 | 4 |
|  | ψ (litter) *p* (gardening activities) | 300.438 | 0.3565 | 0.09825 | 4 |
|  | ψ (litter) *p* (tree and shrub richness) | 301.135 | 1.0535 | 0.06934 | 4 |
|  | ψ (tree richness) *p* (tree richness) | 301.8673 | 1.7858 | 0.04808 | 4 |
| Cold-Dry 2016 | ψ (litter) *p* (shrub cover) | 252.3047 | 0 | 0.28298 | 4 |
| Warm-Dry 2016 | ψ (tree and shrub richness) *p* (tree and shrub richness) | 363.8335 | 0 | 0.06718 | 4 |
| Rainy 2016 | ψ (shrub cover) *p* (intercept only) | 640.7659 | 0 | 0.05442 | 3 |
|  | ψ (shrub cover) *p* (herb cover) | 641.1409 | 0.375 | 0.04512 | 4 |
|  | ψ (shrub cover) *p* (gardening activities) | 641.2603 | 0.4944 | 0.0425 | 4 |
|  | ψ (tree cover) *p* (gardening activities) | 641.8844 | 1.1185 | 0.03111 | 4 |
|  | ψ (shrub cover) *p* (distance to urban structure) | 642.4728 | 1.7069 | 0.02318 | 4 |
|  | ψ (shrub cover) *p* (tree richness) | 642.4817 | 1.7158 | 0.02308 | 4 |
|  | ψ (shrub cover) *p* (tree and shrub richness) | 642.5046 | 1.7387 | 0.02282 | 4 |
|  | ψ (tree cover) *p* (intercept only) | 642.7351 | 1.9692 | 0.02033 | 3 |
|  | ψ (shrub cover) *p* (tree cover) | 642.7542 | 1.9883 | 0.02014 | 4 |
| Cold-Dry 2017 | ψ (distance to urban structure) *p* (intercept only) | 387.1486 | 0 | 0.04628 | 3 |
|  | ψ (distance to urban structure) *p* (tree richness) | 387.1607 | 0.0121 | 0.046 | 4 |
|  | ψ (intercept only) *p* (distance to urban structure) | 387.3756 | 0.227 | 0.04132 | 3 |
|  | ψ (distance to urban structure) *p* (herb cover) | 387.8945 | 0.7459 | 0.03187 | 4 |
|  | ψ (distance to urban structure) *p* (gardening activities) | 388.249 | 1.1004 | 0.0267 | 4 |
|  | ψ (distance to urban structure) *p* (exposed rock) | 388.3182 | 1.1696 | 0.02579 | 4 |
|  | ψ (litter) *p* (distance to urban structure) | 388.3332 | 1.1846 | 0.0256 | 4 |
|  | ψ (distance to urban structure) *p* (tree and shrub richness) | 388.3541 | 1.2055 | 0.02533 | 4 |
|  | ψ (tree and shrub richness) *p* (distance to urban structure) | 388.7759 | 1.6273 | 0.02051 | 4 |
|  | ψ (distance to urban structure) *p* (tree cover) | 388.9709 | 1.8223 | 0.01861 | 4 |
|  | ψ (exposed rock) *p* (distance to urban structure) | 388.9915 | 1.8429 | 0.01842 | 4 |
|  | ψ (gardening activities) *p* (distance to urban structure) | 389.0108 | 1.8622 | 0.01824 | 4 |
|  | ψ (distance to urban structure) *p* (distance to urban structure) | 389.0773 | 1.9287 | 0.01764 | 4 |
| Mexica red-bellied squirrel (*Sciurus aureogaster*) |  |  |  |  |  |
| Warm-Dry 2015 | ψ (tree cover) *p* (gardening activities) | 305.2269 | 0 | 0.19639 | 4 |
|  | ψ (shrub cover) *p* (gardening activities) | 306.9742 | 1.7473 | 0.08198 | 4 |
| Rainy 2015 | ψ (herb cover) *p* (distance to urban structure) | 192.0788 | 0 | 0.10191 | 4 |
|  | ψ (type of area) *p* (herb cover) | 193.3559 | 1.2771 | 0.05381 | 4 |
|  | ψ (herb cover) *p* (type of area) | 193.4317 | 1.3529 | 0.05181 | 4 |
|  | ψ (type of area) *p* (distance to urban structure) | 193.4946 | 1.4158 | 0.05021 | 4 |
|  | ψ (litter) *p* (distance to urban structure) | 193.613 | 1.5342 | 0.04732 | 4 |
|  | ψ (intercept only) *p* (distance to urban structure) | 193.6378 | 1.559 | 0.04674 | 3 |
|  | ψ (tree richness) *p* (type of area) | 193.7147 | 1.6359 | 0.04498 | 4 |
|  | ψ (tree richness) *p* (distance to urban structure) | 193.7378 | 1.659 | 0.04446 | 4 |
|  | ψ (distance to urban structure) *p* (distance to urban structure) | 193.7772 | 1.6984 | 0.04359 | 4 |
| Cold-Dry 2016 | ψ (shrub cover) *p* (gardening activities) | 264.8248 | 0 | 0.11568 | 4 |
|  | ψ (type of area) *p* (shrub cover) | 265.911 | 1.0862 | 0.06721 | 4 |
|  | ψ (shrub cover) *p* (type of area) | 266.1292 | 1.3044 | 0.06026 | 4 |
|  | ψ (type of area) *p* (gardening activities) | 266.3371 | 1.5123 | 0.05431 | 4 |
|  | ψ (gardening activities) *p* (shrub cover) | 266.447 | 1.6222 | 0.05141 | 4 |
|  | ψ (shrub cover) *p* (herb cover) | 266.6922 | 1.8674 | 0.04548 | 4 |
| Warm-Dry 2016 | ψ (tree richness) *p* (herb cover) | 283.7284 | 0 | 0.36811 | 4 |
|  | ψ (tree richness) *p* (shrub cover) | 285.0405 | 1.3121 | 0.19101 | 4 |
| Rainy 2016 | ψ (distance to urban structure) *p* (shrub cover) | 461.1537 | 0 | 0.2353 | 4 |
| Cold-Dry 2017 | ψ (tree richness) *p* (herb cover) | 356.9055 | 0 | 0.52132 | 4 |
| Rock squirrel (*Otospermophilus variegatus*) |  |  |  |  |  |
| Warm-Dry 2015 | ψ (tree cover) *p* (gardening activities) | 282.3922 | 0 | 0.25097 | 4 |
|  | ψ (gardening activities) *p* (tree cover) | 283.3698 | 0.9776 | 0.15394 | 4 |
| Rainy 2015 | ψ (shrub cover) *p* (shrub cover) | 124.2376 | 0 | 0.13189 | 4 |
|  | ψ (herb cover) *p* (tree cover) | 124.3344 | 0.0968 | 0.12566 | 4 |
|  | ψ (litter) *p* (shrub cover) | 124.582 | 0.3444 | 0.11103 | 4 |
|  | ψ (exposed rock) *p* (shrub cover) | 125.8515 | 1.6139 | 0.05885 | 4 |
| Cold-Dry 2016 | ψ (litter) *p* (shrub cover) | 99.8021 | 0 | 0.05882 | 4 |
|  | ψ (gardening activities) *p* (shrub cover) | 99.9433 | 0.1412 | 0.05481 | 3 |
|  | ψ (shrub cover) *p* (type of area) | 100.0134 | 0.2113 | 0.05292 | 4 |
|  | ψ (gardening activities) *p* (type of area) | 100.0441 | 0.242 | 0.05212 | 4 |
|  | ψ (tree cover) *p* (type of area) | 100.4305 | 0.6284 | 0.04296 | 4 |
|  | ψ (herb cover) *p* (gardening activities) | 100.985 | 1.1829 | 0.03256 | 3 |
|  | ψ (gardening activities) *p* (tree and shrub richness) | 100.9889 | 1.1868 | 0.03249 | 3 |
|  | ψ (tree and shrub richness) *p* (gardening activities) | 101.0858 | 1.2837 | 0.03096 | 3 |
|  | ψ (gardening activities) *p* (exposed rock) | 101.1183 | 1.3162 | 0.03046 | 3 |
|  | ψ (tree richness) *p* (gardening activities) | 101.1312 | 1.3291 | 0.03026 | 3 |
|  | ψ (gardening activities) *p* (gardening activities) | 101.1725 | 1.3704 | 0.02964 | 3 |
|  | ψ (shrub cover) *p* (intercept only) | 101.2083 | 1.4062 | 0.02912 | 3 |
|  | ψ (herb cover) *p* (type of area) | 101.5656 | 1.7635 | 0.02435 | 4 |
|  | ψ (tree and shrub richness) *p* (type of area) | 101.6347 | 1.8326 | 0.02353 | 4 |
|  | ψ (tree richness) *p* (type of area) | 101.7252 | 1.9231 | 0.02249 | 4 |
| Warm-Dry 2016 | ψ (exposed rock) *p* (distance to urban structure) | 181.0333 | 0 | 0.08743 | 4 |
|  | ψ (intercept only) *p* (gardening activities) | 181.2369 | 0.2036 | 0.07897 | 2 |
|  | ψ (gardening activities) *p* (intercept only) | 181.2369 | 0.2036 | 0.07897 | 2 |
|  | ψ (gardening activities) *p* (tree and shrub richness) | 181.7538 | 0.7205 | 0.06098 | 3 |
|  | ψ (gardening activities) *p* (exposed rock) | 181.7568 | 0.7235 | 0.06089 | 4 |
|  | ψ (gardening activities) *p* (tree cover) | 182.3111 | 1.2778 | 0.04615 | 3 |
|  | ψ (tree and shrub richness) *p* (gardening activities) | 182.5146 | 1.4813 | 0.04169 | 3 |
|  | ψ (gardening activities) *p* (tree richness) | 182.7468 | 1.7135 | 0.03712 | 3 |
| Rainy 2016 | ψ (herb cover) *p* (type of area) | 202.162 | 0 | 0.12064 | 5 |
|  | ψ (type of area) *p* (type of area) | 203.6067 | 1.4447 | 0.05858 | 4 |
|  | ψ (intercept only) *p* (type of area) | 203.6601 | 1.4981 | 0.05704 | 4 |
|  | ψ (distance to urban structure) *p* (distance to urban structure) | 204.0261 | 1.8641 | 0.0475 | 4 |
| Cold-Dry 2017 | ψ (exposed rock) *p* (tree richness) | 71.2474 | 0 | 0.3907 | 4 |
|  | ψ (exposed rock) *p* (type of area) | 72.5747 | 1.3273 | 0.2012 | 3 |
| Rose natal grass (*Melinis repens*) |  |  |  |  |  |
| Warm-Dry 2015 | ψ (tree richness) *p* (exposed rock) | 718.9402 | 0 | 0.11671 | 4 |
|  | ψ (intercept only) *p* (exposed rock) | 719.0312 | 0.091 | 0.11152 | 3 |
|  | ψ (tree and shrub richness) *p* (exposed rock) | 719.0944 | 0.1542 | 0.10805 | 4 |
|  | ψ (herb cover) *p* (exposed rock) | 719.1096 | 0.1694 | 0.10723 | 4 |
|  | ψ (tree richness) *p* (tree cover) | 719.8119 | 0.8717 | 0.07548 | 3 |
|  | ψ (tree cover) *p* (exposed rock) | 720.0039 | 1.0637 | 0.06857 | 4 |
|  | ψ (exposed rock) *p* (exposed rock) | 720.4827 | 1.5425 | 0.05397 | 4 |
|  | ψ (shrub cover) *p* (exposed rock) | 720.9042 | 1.964 | 0.04372 | 4 |
| Rainy 2015 | ψ (shrub cover) *p* (tree cover) | 448.7219 | 0 | 0.30564 | 4 |
|  | ψ (shrub cover) *p* (distance to urban structure) | 448.8902 | 0.1683 | 0.28097 | 4 |
|  | ψ (shrub cover) *p* (exposed rock) | 449.0291 | 0.3072 | 0.26212 | 4 |
| Cold-Dry 2016 | ψ (shrub cover) *p* (gardening activities) | 571.2422 | 0 | 0.28468 | 4 |
|  | ψ (gardening activities) *p* (type of area) | 571.8726 | 0.6304 | 0.20771 | 4 |
| Warm-Dry 2016 | ψ (shrub cover) *p* (shrub cover) | 498.1979 | 0 | 0.6435 | 4 |
| Rainy 2016 | ψ (shrub cover) *p* (exposed rock) | 632.8302 | 0 | 0.70641 | 4 |
| Cold-Dry 2017 | ψ (shrub cover) *p* (tree cover) | 543.0649 | 0 | 0.74975 | 4 |
| Kikuyo grass (*Pennisetum clandestinum*) |  |  |  |  |  |
| Warm-Dry 2015 | ψ (shrub cover) *p* (distance to urban structure) | 702.6107 | 0 | 0.6868 | 4 |
| Rainy 2015 | ψ (shrub cover) *p* (type of area) | 410.5676 | 0 | 0.90309 | 5 |
| Cold-Dry 2016 | ψ (shrub cover) *p* (gardening activities) | 571.2422 | 0 | 0.28468 | 4 |
|  | ψ (gardening activities) *p* (type of area) | 571.8726 | 0.6304 | 0.20771 | 4 |
| Warm-Dry 2016 | ψ (shrub cover) *p* (herb cover) | 613.2846 | 0 | 0.57298 | 4 |
|  | ψ (type of area) *p* (herb cover) | 614.056 | 0.7714 | 0.38961 | 4 |
| Rainy 2016 | ψ (shrub cover) *p* (shrub cover) | 855.4239 | 0 | 0.16644 | 4 |
|  | ψ (gardening activities) *p* (type of area) | 856.1247 | 0.7008 | 0.11724 | 4 |
|  | ψ (type of area) *p* (exposed rock) | 856.1527 | 0.7288 | 0.11561 | 3 |
|  | ψ (soil depth) *p* (shrub cover) | 856.1859 | 0.762 | 0.11371 | 4 |
|  | ψ (shrub cover) *p* (gardening activities) | 856.8025 | 1.3786 | 0.08354 | 4 |
|  | ψ (type of area) *p* (tree and shrub richness) | 856.8155 | 1.3916 | 0.083 | 3 |
|  | ψ (type of area) *p* (intercept only) | 857.011 | 1.5871 | 0.07527 | 3 |
|  | ψ (distance to urban structure) *p* (shrub cover) | 857.1309 | 1.707 | 0.07089 | 4 |
| Cold-Dry 2017 | ψ (gardening activities) *p* (shrub cover) | 750.2712 | 0 | 0.66998 | 4 |
| Muhly grass (*Muhlenbergia robusta*) |  |  |  |  |  |
| Warm-Dry 2015 | ψ (distance to urban structure) *p* (shrub cover) | 508.1324 | 0 | 0.32247 | 4 |
| Rainy 2015 | ψ (gardening activities) *p* (type of area) | 508.8122 | 0.6798 | 0.22955 | 5 |
| Cold-Dry 2016 | ψ (soil depth) *p* (shrub cover) | 509.1643 | 1.0319 | 0.19249 | 4 |
| Warm-Dry 2016 | ψ (shrub cover) *p* (distance to urban structure) | 402.9535 | 0 | 0.96034 | 4 |
| Rainy 2016 | ψ (shrub cover) *p* (distance to urban structure) | 441.973 | 0 | 0.70623 | 4 |
| Cold-Dry 2017 | ψ (shrub cover) *p* (shrub cover) | 463.198 | 0 | 0.69642 | 4 |
|  | ψ (shrub cover) *p* (distance to urban structure) | 539.1775 | 0 | 0.91876 | 4 |
|  | ψ (shrub cover) *p* (shrub cover) | 510.2591 | 0 | 0.99468 | 4 |
| Peruvian pepper (*Schinus molle*) |  |  |  |  |  |
| Warm-Dry 2015 | ψ (tree and shrub richness) *p* (type of area) | 652.6611 | 0 | 0.42856 | 5 |
|  | ψ (tree and shrub richness) *p* (gardening activities) | 654.5738 | 1.9127 | 0.16469 | 4 |
| Rainy 2015 | ψ (type of area) *p* (exposed rock) | 639.9649 | 0 | 0.08786 | 5 |
|  | ψ (tree richness) *p* (exposed rock) | 641.5461 | 1.5812 | 0.03985 | 4 |
| Cold-Dry 2016 | ψ (tree and shrub richness) *p* (type of area) | 615.5036 | 0 | 0.85344 | 5 |
| Warm-Dry 2016 | ψ (tree and shrub richness) *p* (gardening activities) | 595.3795 | 0 | 0.18713 | 4 |
|  | ψ (tree and shrub richness) *p* (intercept only) | 596.2472 | 0.8677 | 0.12126 | 3 |
|  | ψ (tree and shrub richness) *p* (exposed rock) | 596.8262 | 1.4467 | 0.09078 | 4 |
|  | ψ (tree and shrub richness) *p* (distance to urban structure) | 596.9552 | 1.5757 | 0.08511 | 4 |
| Rainy 2016 | ψ (gardening activities) *p* (tree and shrub richness) | 834.7358 | 0 | 0.22849 | 4 |
|  | ψ (type of area) *p* (tree and shrub richness) | 835.0825 | 0.3467 | 0.19213 | 5 |
|  | ψ (shrub cover) *p* (tree and shrub richness) | 836.2491 | 1.5133 | 0.10722 | 4 |
|  | ψ (distance to urban structure) *p* (tree and shrub richness) | 836.498 | 1.7622 | 0.09467 | 4 |
| Cold-Dry 2017 | ψ (tree and shrub richness) *p* (gardening activities) | 733.8384 | 0 | 0.08354 | 4 |
|  | ψ (tree and shrub richness) *p* (intercept only) | 735.271 | 1.4326 | 0.04081 | 3 |
|  | ψ (tree and shrub richness) *p* (herb cover) | 735.3605 | 1.5221 | 0.03903 | 4 |
|  | ψ (tree and shrub richness) *p* (tree cover) | 735.6113 | 1.7729 | 0.03443 | 4 |
| Tepozan tree (*Buddleia cordata*) |  |  |  |  |  |
| Warm-Dry 2015 | ψ (shrub cover) *p* (shrub cover) | 516.2334 | 0 | 0.8866 | 4 |
| Rainy 2015 | ψ (shrub cover) *p* (shrub cover) | 490.8545 | 0 | 0.76361 | 4 |
| Cold-Dry 2016 | ψ (tree and shrub richness) *p* (type of area) | 512.03 | 0 | 0.27011 | 5 |
|  | ψ (tree and shrub richness) *p* (tree richness) | 512.2889 | 0.2589 | 0.23732 | 4 |
|  | ψ (tree and shrub richness) *p* (distance to urban structure) | 512.787 | 0.757 | 0.185 | 4 |
| Warm-Dry 2016 | ψ (tree and shrub richness) *p* (type of area) | 524.6835 | 0 | 0.46699 | 5 |
|  | ψ (tree and shrub richness) *p* (distance to urban structure) | 525.2965 | 0.613 | 0.34371 | 4 |
| Rainy 2016 | ψ (tree and shrub richness) *p* (shrub cover) | 680.0647 | 0 | 0.64246 | 4 |
| Cold-Dry 2017 | ψ (shrub cover) *p* (type of area) | 634.604 | 0 | 0.53581 | 5 |
|  | ψ (shrub cover) *p* (shrub cover) | 636.5147 | 1.9107 | 0.20611 | 4 |
| River red gum (*Eucaliptus camaldulensis*) |  |  |  |  |  |
| Warm-Dry 2015 | ψ (distance to urban structure) *p* (herb cover) | 511.3342 | 0 | 0.63281 | 4 |
| Rainy 2015 | ψ (distance to urban structure) *p* (tree and shrub richness) | 404.7729 | 0 | 0.46919 | 4 |
|  | ψ (distance to urban structure) *p* (tree cover) | 405.9228 | 1.1499 | 0.26403 | 4 |
| Cold-Dry 2016 | ψ (distance to urban structure) *p* (tree richness) | 455.8635 | 0 | 0.86547 | 4 |
| Warm-Dry 2016 | ψ (distance to urban structure) *p* (type of area) | 551.3817 | 0 | 0.32008 | 5 |
|  | ψ (tree and shrub richness) *p* (type of area) | 551.6463 | 0.2646 | 0.28041 | 5 |
| Rainy 2016 | ψ (distance to urban structure) *p* (tree richness) | 677.1333 | 0 | 0.99242 | 4 |
| Cold-Dry 2017 | ψ (distance to urban structure) *p* (type of area) | 637.6463 | 0 | 0.22452 | 5 |
|  | ψ (distance to urban structure) *p* (tree cover) | 637.973 | 0.3267 | 0.19068 | 4 |
| Tropical ash (*Fraxinus udhei*) |  |  |  |  |  |
| Warm-Dry 2015 | ψ (shrub cover) *p* (gardening activities) | 518.8996 | 0 | 0.2415 | 4 |
|  | ψ (type of area) *p* (gardening activities) | 519.04 | 0.1404 | 0.22513 | 5 |
|  | ψ (shrub cover) *p* (type of area) | 519.8379 | 0.9383 | 0.15107 | 5 |
| Rainy 2015 | ψ (exposed rock) *p* (type of area) | 463.8907 | 0 | 0.34864 | 5 |
|  | ψ (shrub cover) *p* (type of area) | 465.254 | 1.3633 | 0.17634 | 5 |
| Cold-Dry 2016 | ψ (type of area) *p* (shrub cover) | 459.5053 | 0 | 0.42944 | 5 |
| Warm-Dry 2016 | ψ (tree richness) *p* (type of area) | 605.8353 | 0 | 0.28756 | 5 |
|  | ψ (shrub cover) *p* (exposed rock) | 606.7595 | 0.9242 | 0.18115 | 4 |
|  | ψ (type of area) *p* (exposed rock) | 607.7808 | 1.9455 | 0.10871 | 5 |
| Rainy 2016 | ψ (type of area) *p* (shrub cover) | 691.0153 | 0 | 0.47452 | 5 |
|  | ψ (type of area) *p* (gardening activities) | 692.2628 | 1.2475 | 0.25431 | 5 |
| Cold-Dry 2017 | ψ (gardening activities) *p* (shrub cover) | 736.0006 | 0 | 0.16938 | 4 |
|  | ψ (distance to urban structure) *p* (shrub cover) | 736.0644 | 0.0638 | 0.16406 | 4 |
|  | ψ (tree cover) *p* (shrub cover) | 736.1163 | 0.1157 | 0.15986 | 4 |
|  | ψ (soil depth) *p* (shrub cover) | 736.7985 | 0.7979 | 0.11366 | 4 |
|  | ψ (type of area) *p* (shrub cover) | 737.6848 | 1.6842 | 0.07297 | 5 |
| Grey silky-flycatcher (*Ptiliogonys cinereus*) |  |  |  |  |  |
| Warm-Dry 2015 | ψ (distance to urban structure) *p* (tree cover) | 262.0467 | 0 | 0.14921 | 4 |
|  | ψ (gardening activities) *p* (type of area) | 262.6923 | 0.6456 | 0.10804 | 4 |
|  | ψ (type of area) *p* (gardening activities) | 262.7689 | 0.7222 | 0.10398 | 4 |
|  | ψ (distance to urban structure) *p* (type of area) | 263.1945 | 1.1478 | 0.08405 | 4 |
|  | ψ (type of area) *p* (shrub cover) | 263.5815 | 1.5348 | 0.06926 | 4 |
|  | ψ (type of area) *p* (type of area) | 263.7397 | 1.693 | 0.064 | 4 |
|  | ψ (tree richness) *p* (type of area) | 263.8295 | 1.7828 | 0.06119 | 4 |
|  | ψ (herb cover) *p* (type of area) | 263.9057 | 1.859 | 0.0589 | 4 |
|  | ψ (litter) *p* (type of area) | 263.9136 | 1.8669 | 0.05867 | 4 |
| Rainy 2015 | ψ (shrub cover) *p* (distance to urban structure) | 229.4371 | 0 | 0.134 | 4 |
|  | ψ (tree and shrub richness) *p* (distance to urban structure) | 231.0153 | 1.5782 | 0.06087 | 4 |
| Cold-Dry 2016 | ψ (intercept only) *p* (shrub cover) | 241.9566 | 0 | 0.03309 | 3 |
|  | ψ (intercept only) *p* (exposed rock) | 242.6288 | 0.6722 | 0.02365 | 3 |
|  | ψ (intercept only) *p* (intercept only) | 242.644 | 0.6874 | 0.02347 | 2 |
|  | ψ (distance to urban structure) *p* (shrub cover) | 242.7083 | 0.7517 | 0.02272 | 4 |
|  | ψ (intercept only) *p* (herb cover) | 242.8027 | 0.8461 | 0.02168 | 3 |
|  | ψ (intercept only) *p* (tree and shrub richness) | 242.8753 | 0.9187 | 0.0209 | 3 |
|  | ψ (intercept only) *p* (tree richness) | 243.3531 | 1.3965 | 0.01646 | 3 |
|  | ψ (herb cover) *p* (shrub cover) | 243.683 | 1.7264 | 0.01396 | 4 |
|  | ψ (tree richness) *p* (shrub cover) | 243.7012 | 1.7446 | 0.01383 | 4 |
|  | ψ (tree richness) *p* (exposed rock) | 243.717 | 1.7604 | 0.01372 | 4 |
|  | ψ (tree and shrub richness) *p* (shrub cover) | 243.7491 | 1.7925 | 0.01351 | 4 |
|  | ψ (distance to urban structure) *p* (intercept only) | 243.8188 | 1.8622 | 0.01304 | 3 |
|  | ψ (tree and shrub richness) *p* (tree and shrub richness) | 243.8359 | 1.8793 | 0.01293 | 4 |
|  | ψ (distance to urban structure) *p* (herb cover) | 243.852 | 1.8954 | 0.01283 | 4 |
|  | ψ (distance to urban structure) *p* (tree and shrub richness) | 243.9118 | 1.9552 | 0.01245 | 4 |
|  | ψ (gardening activities) *p* (shrub cover) | 243.9557 | 1.9991 | 0.01218 | 4 |
| Warm-Dry 2016 | ψ (distance to urban structure) *p* (herb cover) | 475.3584 | 0 | 0.14616 | 4 |
|  | ψ (distance to urban structure) *p* (tree and shrub richness) | 476.4 | 1.0416 | 0.08683 | 4 |
|  | ψ (distance to urban structure) *p* (exposed rock) | 476.5257 | 1.1673 | 0.08154 | 4 |
|  | ψ (distance to urban structure) *p* (intercept only) | 476.6441 | 1.2857 | 0.07685 | 3 |
| Rainy 2016 | ψ (gardening activities) *p* (distance to urban structure) | 369.7777 | 0 | 0.39085 | 4 |
| Cold-Dry 2017 | ψ (distance to urban structure) *p* (tree richness) | 415.8378 | 0 | 0.79034 | 4 |
| Bushtit (*Psaltriparus minimus*) |  |  |  |  |  |
| Warm-Dry 2015 | ψ (distance to urban structure) *p* (distance to urban structure) | 419.705 | 0 | 0.28087 | 4 |
| Rainy 2015 | ψ (tree and shrub richness) *p* (distance to urban structure) | 301.9681 | 0 | 0.06923 | 4 |
|  | ψ (intercept only) *p* (distance to urban structure) | 302.3167 | 0.3486 | 0.05815 | 3 |
|  | ψ (tree richness) *p* (distance to urban structure) | 303.1549 | 1.1868 | 0.03824 | 4 |
|  | ψ (gardening activities) *p* (distance to urban structure) | 303.3168 | 1.3487 | 0.03527 | 4 |
| Cold-Dry 2016 | ψ (intercept only) *p* (gardening activities) | 148.9872 | 0 | 0.16436 | 3 |
|  | ψ (tree richness) *p* (gardening activities) | 149.7901 | 0.8029 | 0.11001 | 4 |
|  | ψ (distance to urban structure) *p* (gardening activities) | 149.8445 | 0.8573 | 0.10706 | 4 |
|  | ψ (tree cover) *p* (gardening activities) | 150.1682 | 1.181 | 0.09106 | 4 |
|  | ψ (tree and shrub richness) *p* (gardening activities) | 150.5696 | 1.5824 | 0.0745 | 4 |
|  | ψ (gardening activities) *p* (gardening activities) | 150.7207 | 1.7335 | 0.06908 | 4 |
| Warm-Dry 2016 | ψ (distance to urban structure) *p* (shrub cover) | 354.333 | 0 | 0.35759 | 4 |
| Rainy 2016 | ψ (gardening activities) *p* (intercept only) | 447.6173 | 0 | 0.1008 | 3 |
|  | ψ (type of area) *p* (intercept only) | 448.7065 | 1.0892 | 0.05847 | 4 |
|  | ψ (gardening activities) *p* (tree and shrub richness) | 449.1366 | 1.5193 | 0.04716 | 4 |
|  | ψ (gardening activities) *p* (shrub cover) | 449.1877 | 1.5704 | 0.04597 | 4 |
|  | ψ (gardening activities) *p* (tree cover) | 449.1916 | 1.5743 | 0.04588 | 4 |
|  | ψ (gardening activities) *p* (distance to urban structure) | 449.4212 | 1.8039 | 0.0409 | 4 |
|  | ψ (gardening activities) *p* (exposed rock) | 449.4544 | 1.8371 | 0.04023 | 4 |
| Cold-Dry 2017 | ψ (distance to urban structure) *p* (distance to urban structure) | 282.9763 | 0 | 0.28325 | 4 |
|  | ψ (tree and shrub richness) *p* (distance to urban structure) | 283.6661 | 0.6898 | 0.20062 | 4 |

For each species and for each season we show models with strong support (ΔAICc < 2).

*w* = relative support for each model in the data (Akaike weights). *k* = number of parameters.
