# Supplementary material for: This town ain't big enough for both of us…or is it? Spatial co-occurrence between exotic and native species in an urban reserve
Source: PLoS One. 2019 Jan 18;14(1):e0211050. doi: 10.1371/journal.pone.0211050 (PMC6338412; doi:10.1371/journal.pone.0211050)
Supplement: S2 Table — To avoid the double counting of animal individuals in the field, counts were made by a single observer within the first five minutes of the observation period. *In the case of grasses, we could not count individuals. (DOCX) [file pone.0211050.s002.docx]

**S2 Table. Number of sites where the study species were detected and their relative abundances estimated as the average number of detected individuals across six sampling periods.**

| **Exotic or native** | **Study species** | **Number of sites where the species was detected** | | | | | | **Average number of observed individuals per season** |
| --- | --- | --- | --- | --- | --- | --- | --- | --- |
|  |  | **Warm-dry 2015** | **Rainy 2015** | **Cold-dry 2016** | **Warm-dry 2016** | **Rainy 2016** | **Cold-dry 2017** |  |
| Exotic species | House sparrow  (*Passer domesticus*) | 67 | 45 | 92 | 75 | 61 | 51 | 104 |
|  | Mexican red-bellied squirrel  (*Sciurus aureogaster*) | 26 | 17 | 23 | 25 | 38 | 30 | 26 |
|  | Rose natal grass  (*Melinis repens*) | 91 | 51 | 92 | 53 | 57 | 52 | * |
|  | Kikuyu grass  (*Pennisetum clandestinum*) | 85 | 85 | 92 | 84 | 89 | 82 | * |
|  | Peruvian pepper  (*Schinus molle*) | 79 | 74 | 77 | 78 | 81 | 83 | 122 |
|  | River red gum  (*Eucalyptus camaldulensis*) | 55 | 49 | 52 | 60 | 62 | 63 | 115 |
|  | Tropical ash  (*Fraxinus uhdei*) | 59 | 52 | 44 | 64 | 62 | 78 | 212 |
| Native  species | House finch  (*Haemorhous mexicanus*) | 72 | 58 | 55 | 79 | 93 | 66 | 92 |
|  | Inca dove  (*Columbina inca*) | 36 | 30 | 20 | 31 | 39 | 23 | 23 |
|  | American robin  (*Turdus migratorius*) | 45 | 15 | 17 | 51 | 18 | 25 | 26 |
|  | Bewick's wren  (*Thryomanes bewickii*) | 27 | 31 | 25 | 37 | 62 | 35 | 19 |
|  | Rock squirrel  (*Otospermophilus variegatus*) | 29 | 11 | 7 | 15 | 18 | 5 | 14 |
|  | Muhly grass  (*Muhlenbergia robusta*) | 57 | 49 | 57 | 58 | 58 | 59 | * |
|  | Tepozan tree  (*Buddleja cordata*) | 85 | 86 | 84 | 85 | 87 | 85 | 310 |
|  | Grey silky-flycatcher  (*Ptiliogonys cinereus*) | 22 | 20 | 20 | 44 | 30 | 36 | 32 |
|  | Bushtit  (*Psaltriparus minimus*) | 39 | 28 | 12 | 31 | 37 | 24 | 64 |

To avoid the double counting of animal individuals in the field, counts were made by a single observer within the first five minutes of the observation period.

*In the case of grasses, we could not count individuals.
