# Supplementary material for: This town ain't big enough for both of us…or is it? Spatial co-occurrence between exotic and native species in an urban reserve
Source: PLoS One. 2019 Jan 18;14(1):e0211050. doi: 10.1371/journal.pone.0211050 (PMC6338412; doi:10.1371/journal.pone.0211050)
Supplement: S3 Table — Complete model selection results for two-species occupancy models that tested the hypothesis that the presence of exotic species (denoted as A) influences the occupancy of native species (denoted as B). For each pair of exotic-native species and for each season we used the Akaike’s information criterion adjusted for small sample sizes (AICc) to compare a model in which the occupancy (ψ) of the native species depends on the presence (ψBA) or absence (ψBa) of the exotic species against a model in which the occupancy of the native species is independent of the presence of the exotic species (ψB). The environmental covariates with strongest effect on the parameters are shown within parentheses. (DOCX) [file pone.0211050.s003.docx]

**S3 Table. Complete model selection results for two-species occupancy models that tested the hypothesis that the presence of exotic species (denoted as A) influences the occupancy of native species (denoted as B).**

|  | **Season and year** | **Model** | **AICc** | **ΔAICc** | ***w*** | ***k*** |
| --- | --- | --- | --- | --- | --- | --- |
| A – House sparrow  (*Passer domesticus*) | Warm-dry 2015 | ψ^A^ (gardening activities) ψ^B^ (shrub cover) *p*^A^ (distance to urban structure) *p*^B^ (type of area) | 1186.34 | 0.00 | 0.92 | 9 |
| B – House finch  (*Haemorhous mexicanus*) |  | ψ^A^ (gardening activities) ψ^BA^ (shrub cover) ψ^Ba^ (shrub cover) *p*^A^ (distance to urban structure) *p*^B^ (type of area) | 1191.24 | 4.90 | 0.08 | 11 |
|  |  |  |  |  |  |  |
|  | Rainy 2015 | ψ^A^ (shrub cover) ψ^B^ (type of area) *p*^A^ (distance to urban structure) *p*^B^ (herb cover) | 937.30 | 0.00 | 0.94 | 9 |
|  |  | ψ^A^ (shrub cover) ψ^BA^ (type of area) ψ^Ba^ (type of area) *p*^A^ (distance to urban structure) *p*^B^ (herb cover) | 942.99 | 5.69 | 0.06 | 12 |
|  |  |  |  |  |  |  |
|  | Cold-dry 2016 | ψ^A^ (shrub cover) ψ^BA^ (shrub cover) ψ^Ba^ (shrub cover) *p*^A^ (gardening activities) *p*^B^ (shrub cover) | 1124.55 | 0.00 | 0.59 | 10 |
|  |  | ψ^A^ (shrub cover) ψ^B^ (shrub cover) *p*^A^ (gardening activities) *p*^B^ (shrub cover) | 1125.29 | 0.74 | 0.41 | 8 |
|  |  |  |  |  |  |  |
|  | Warm-dry 2016 | ψ^A^ (distance to urban structure) ψ^B^ (intercept only) *p*^A^ (type of area) *p*^B^ (shrub cover) | 1293.43 | 0.00 | 0.68 | 8 |
|  |  | ψ^A^ (distance to urban structure) ψ^BA^ (intercept only) ψ^Ba^ (intercept only) *p*^A^ (type of area) *p*^B^ (shrub cover) | 1294.93 | 1.51 | 0.32 | 9 |
|  |  |  |  |  |  |  |
|  | Rainy 2016 | ψ^A^ (type of area) ψ^B^ (intercept only) *p*^A^ (shrub cover) *p*^B^ (distance to urban structure) | 1602.47 | 0.00 | 0.71 | 8 |
|  |  | ψ^A^ (type of area) ψ^BA^ (intercept only) ψ^Ba^ (intercept only) *p*^A^ (shrub cover) *p*^B^ (distance to urban structure) | 1604.28 | 1.81 | 0.29 | 9 |
|  |  |  |  |  |  |  |
|  | Cold-dry 2017 | ψ^A^ (distance to urban structure) ψ^BA^ (intercept only) ψ^Ba^ (intercept only) *p*^A^ (shrub cover) *p*^B^ (distance to urban structure) | 1185.27 | 0.00 | 0.98 | 8 |
|  |  | ψ^A^ (distance to urban structure) ψ^B^ (intercept only) *p*^A^ (shrub cover) *p*^B^ (distance to urban structure) | 1193.43 | 8.16 | 0.02 | 7 |
|  |  |  |  |  |  |  |
| A – House sparrow  (*Passer domesticus*) | Warm-dry 2015 | ψ^A^ (gardening activities) ψ^B^ (shrub cover) *p*^A^ (distance to urban structure) *p*^B^ (distance to urban structure) | 914.70 | 0.00 | 0.70 | 8 |
| B – Inca dove  (*Columbina inca*) |  | ψ^A^ (gardening activities) ψ^BA^ (shrub cover) ψ^Ba^ (shrub cover) *p*^A^ (distance to urban structure) *p*^B^ (distance to urban structure) | 916.37 | 1.66 | 0.30 | 10 |
|  |  |  |  |  |  |  |
|  | Rainy 2015 | ψ^A^ (shrub cover) ψ^B^ (gardening activities) *p*^A^ (distance to urban structure) *p*^B^ (shrub cover) | 721.86 | 0.00 | 0.78 | 8 |
|  |  | ψ^A^ (shrub cover) ψ^BA^ (gardening activities) ψ^Ba^ (gardening activities) *p*^A^ (distance to urban structure) *p*^B^ (shrub cover) | 724.34 | 2.48 | 0.22 | 10 |
|  |  |  |  |  |  |  |
|  | Cold-dry 2016 | ψ^A^ (shrub cover) ψ^B^ (distance to urban structure) *p*^A^ (gardening activities) *p*^B^ (tree and shrub richness) | 818.01 | 0.00 | 0.91 | 8 |
|  |  | ψ^A^ (shrub cover) ψ^BA^ (distance to urban structure) ψ^Ba^ (distance to urban structure) *p*^A^ (gardening activities) *p*^B^ (tree and shrub richness) | 822.66 | 4.65 | 0.09 | 10 |
|  |  |  |  |  |  |  |
|  | Warm-dry 2016 | ψ^A^ (distance to urban structure) ψ^B^ (distance to urban structure) *p*^A^ (type of area) *p*^B^ (exposed rock) | 922.55 | 0.00 | 0.90 | 9 |
|  |  | ψ^A^ (distance to urban structure) ψ^BA^ (distance to urban structure) ψ^Ba^ (distance to urban structure) *p*^A^ (type of area) *p*^B^ (exposed rock) | 926.88 | 4.33 | 0.10 | 11 |
|  |  |  |  |  |  |  |
|  | Rainy 2016 | ψ^A^ (type of area) ψ^BA^ (shrub cover) ψ^Ba^ (shrub cover) *p*^A^ (shrub cover) *p*^B^ (distance to urban structure) | 1129.22 | 0.00 | 0.62 | 11 |
|  |  | ψA(type of area) ψ^B^ (shrub cover) *p*^A^ (shrub cover) *p*^B^ (distance to urban structure) | 1130.17 | 0.95 | 0.38 | 9 |
|  |  |  |  |  |  |  |
|  | Cold-dry 2017 | ψ^A^ (distance to urban structure) ψ^B^ (shrub cover) *p*^A^ (shrub cover) *p*^B^ (tree and shrub richness) | 792.48 | 0.00 | 0.69 | 8 |
|  |  | ψ^A^ (distance to urban structure) ψ^BA^ (shrub cover) ψ^Ba^ (shrub cover) *p*^A^ (shrub cover) *p*^B^ (tree and shrub richness) | 794.07 | 1.59 | 0.31 | 10 |
|  |  |  |  |  |  |  |
| A – House sparrow  (*Passer domesticus*) | Warm-dry 2015 | ψ^A^ (gardening activities) ψ^B^ (type of area) *p*^A^ (distance to urban structure) *p*^B^ (herb cover) | 987.81 | 0.00 | 0.97 | 9 |
| B – American robin  (*Turdus migratorius*) |  | ψ^A^ (gardening activities) ψ^BA^ (type of area) ψ^Ba^ (type of area) *p*^A^ (distance to urban structure) *p*^B^ (herb cover) | 995.04 | 7.23 | 0.03 | 12 |
|  |  |  |  |  |  |  |
|  | Rainy 2015 | ψ^A^ (shrub cover) ψ^B^ (shrub cover) *p*^A^ (distance to urban structure) *p*^B^ (tree richness) | 566.60 | 0.00 | 0.89 | 8 |
|  |  | ψ^A^ (shrub cover) ψ^BA^ (shrub cover) ψ^Ba^ (shrub cover) *p*^A^ (distance to urban structure) *p*^B^ (tree richness) | 570.77 | 4.17 | 0.11 | 10 |
|  |  |  |  |  |  |  |
|  | Cold-dry 2016 | ψ^A^ (shrub cover) ψ^B^ (shrub cover) *p*^A^ (gardening activities) *p*^B^ (exposed rock) | 776.06 | 0.00 | 0.89 | 8 |
|  |  | ψ^A^ (shrub cover) ψ^BA^ (shrub cover) ψ^Ba^ (shrub cover) *p*^A^ (gardening activities) *p*^B^ (exposed rock) | 780.32 | 4.26 | 0.11 | 10 |
|  |  |  |  |  |  |  |
|  | Warm-dry 2016 | ψ^A^ (distance to urban structure) ψ^B^ (type of area) *p*^A^ (type of area) *p*^B^ (herb cover) | 1088.49 | 0.00 | 0.81 | 10 |
|  |  | ψ^A^ (distance to urban structure) ψ^BA^ (type of area) ψ^Ba^ (type of area) *p*^A^ (type of area) *p*^B^ (herb cover) | 1091.39 | 2.90 | 0.19 | 13 |
|  |  |  |  |  |  |  |
|  | Rainy 2016 | ψ^A^ (type of area) ψ^B^ (tree and shrub richness) *p*^A^ (shrub cover) *p*^B^ (shrub cover) | 904.93 | 0.00 | 0.77 | 9 |
|  |  | ψ^A^ (type of area) ψ^BA^ (tree and shrub richness) ψ^Ba^ (tree and shrub richness) *p*^A^ (shrub cover) *p*^B^ (shrub cover) | 907.29 | 2.36 | 0.23 | 11 |
|  |  |  |  |  |  |  |
|  | Cold-dry 2017 | ψ^A^ (distance to urban structure) ψ^B^ (distance to urban structure) *p*^A^ (shrub cover) *p*^B^ (intercept only) | 810.87 | 0.00 | 0.71 | 7 |
|  |  | ψ^A^ (distance to urban structure) ψ^BA^ (distance to urban structure) ψ^Ba^ (distance to urban structure) *p*^A^ (shrub cover) *p*^B^ (intercept only) | 812.61 | 1.74 | 0.29 | 9 |
|  |  |  |  |  |  |  |
| A – House sparrow  (*Passer domesticus*) | Warm-dry 2015 | ψ^A^ (gardening activities) ψ^B^ (tree cover) *p*^A^ (distance to urban structure) *p*^B^ (distance to urban structure) | 818.12 | 0.00 | 0.92 | 8 |
| B – Bewick’s wren  (*Thryomanes bewickii*) |  | ψ^A^ (gardening activities) ψ^BA^ (tree cover) ψ^Ba^ (tree cover) *p*^A^ (distance to urban structure) *p*^B^ (distance to urban structure) | 822.99 | 4.87 | 0.08 | 10 |
|  |  |  |  |  |  |  |
|  | Rainy 2015 | ψ^A^ (shrub cover) ψ^B^ (litter) *p*^A^ (distance to urban structure) *p*^B^ (tree richness) | 699.61 | 0.00 | 0.84 | 8 |
|  |  | ψ^A^ (shrub cover) ψ^BA^ (litter) ψ^Ba^ (litter) *p*^A^ (distance to urban structure) *p*^B^ (tree richness) | 702.88 | 3.28 | 0.16 | 10 |
|  |  |  |  |  |  |  |
|  | Cold-dry 2016 | ψ^A^ (shrub cover) ψ^B^ (litter) *p*^A^ (gardening activities) *p*^B^ (shrub cover) | 824.29 | 0.00 | 0.88 | 8 |
|  |  | ψ^A^ (shrub cover) ψ^BA^ (litter) ψ^Ba^ (litter) *p*^A^ (gardening activities) *p*^B^ (shrub cover) | 828.20 | 3.91 | 0.12 | 10 |
|  |  |  |  |  |  |  |
|  | Warm-dry 2016 | ψ^A^ (distance to urban structure) ψ^B^ (tree and shrub richness) *p*^A^ (type of area) *p*^B^ (tree and shrub richness) | 946.73 | 0.00 | 0.88 | 9 |
|  |  | ψ^A^ (distance to urban structure) ψ^BA^ (tree and shrub richness) ψ^Ba^ (tree and shrub richness) *p*^A^ (type of area) *p*^B^ (tree and shrub richness) | 950.76 | 4.03 | 0.12 | 11 |
|  |  |  |  |  |  |  |
|  | Rainy 2016 | ψ^A^ (type of area) ψ^B^ (shrub cover) *p*^A^ (shrub cover) *p*^B^ (intercept only) | 1297.46 | 0.00 | 0.85 | 8 |
|  |  | ψ^A^ (type of area) ψ^BA^ (shrub cover) ψ^Ba^ (shrub cover) *p*^A^ (shrub cover) *p*^B^ (intercept only) | 1300.92 | 3.46 | 0.15 | 10 |
|  |  |  |  |  |  |  |
|  | Cold-dry 2017 | ψ^A^ (distance to urban structure) ψ^B^ (distance to urban structure) *p*^A^ (shrub cover) *p*^B^ (intercept only) | 901.06 | 0.00 | 0.74 | 7 |
|  |  | ψ^A^ (distance to urban structure) ψ^BA^ (distance to urban structure) ψ^Ba^ (distance to urban structure) *p*^A^ (shrub cover) *p*^B^(intercept only) | 903.11 | 2.05 | 0.26 | 9 |
|  |  |  |  |  |  |  |
| A – Mexican red-bellied squirrel (*Sciurus aureogaster*) | Warm-dry 2015 | ψ^A^ (tree cover) ψ^BA^ (tree cover) ψ^Ba^ (tree cover) *p*^A^ (gardening activities) *p*^B^ (gardening activities) | 581.46 | 0.00 | 0.97 | 10 |
| B – Rock squirrel  (*Otospermophilus variegatus*) |  | ψ^A^ (tree cover) ψ^B^ (tree cover) *p*^A^ (gardening activities) *p*^B^ (gardening activities) | 588.36 | 6.90 | 0.03 | 8 |
|  |  |  |  |  |  |  |
|  | Rainy 2015 | ψ^A^ (herb cover) ψ^B^ (herb cover) *p*^A^ (distance to urban structure) *p*^B^ (tree cover) | 317.15 | 0.00 | 0.90 | 8 |
|  |  | ψ^A^ (herb cover) ψ^BA^ (herb cover) ψ^Ba^ (herb cover) *p*^A^ (distance to urban structure) *p*^B^ (tree cover) | 321.59 | 4.43 | 0.10 | 10 |
|  |  |  |  |  |  |  |
|  | Cold-dry 2016 | ψ^A^ (shrub cover) ψ^B^ (litter) *p*^A^ (gardening activities) *p*^B^ (shrub cover) | 365.37 | 0.00 | 0.89 | 8 |
|  |  | ψ^A^ (shrub cover) ψ^BA^ (litter) ψ^Ba^ (litter) *p*^A^ (gardening activities) *p*^B^ (shrub cover) | 369.64 | 4.28 | 0.11 | 10 |
|  |  |  |  |  |  |  |
|  | Warm-dry 2016 | ψ^A^ (tree richness) ψ^B^ (exposed rock) *p*^A^ (herb cover) *p*^B^ (distance to urban structure) | 465.50 | 0.00 | 0.90 | 8 |
|  |  | ψ^A^ (tree richness) ψ^BA^ (exposed rock) ψ^Ba^ (exposed rock) *p*^A^ (herb cover) *p*^B^ (distance to urban structure) | 469.87 | 4.37 | 0.10 | 10 |
|  |  |  |  |  |  |  |
|  | Rainy 2016 | ψ^A^ (distance to urban structure) ψ^BA^ (herb cover) ψ^Ba^ (herb cover) *p*^A^ (shrub cover) *p*^B^ (type of area) | 653.75 | 0.00 | 0.99 | 11 |
|  |  | ψ^A^ (distance to urban structure) ψ^B^ (herb cover) *p*^A^ (shrub cover) *p*^B^ (type of area) | 664.26 | 10.50 | 0.01 | 9 |
|  |  |  |  |  |  |  |
|  | Cold-dry 2017 | ψ^A^ (tree richness) ψ^BA^ (exposed rock) ψ^Ba^ (exposed rock) *p*^A^ (herb cover) *p*^B^ (tree richness) | 427.82 | 0.00 | 0.63 | 10 |
|  |  | ψ^A^ (tree richness) ψ^B^ (exposed rock) *p*^A^ (herb cover) *p*^B^ (tree richness) | 428.89 | 1.07 | 0.37 | 8 |
|  |  |  |  |  |  |  |
| A – Rose natal grass  (*Melinis repens*) | Warm-dry 2015 | ψ^A^ (intercept only) ψ^B^ (distance to urban structure) *p*^A^ (exposed rock) *p*^B^ (shrub cover) | 1228.58 | 0.00 | 0.68 | 7 |
| B – Muhly grass  (*Muhlenbergia robusta*) |  | ψ^A^ (intercept only) ψ^BA^ (distance to urban structure) ψ^Ba^ (distance to urban structure) *p*^A^ (exposed rock) *p*^B^ (shrub cover) | 1230.10 | 1.52 | 0.32 | 9 |
|  |  |  |  |  |  |  |
|  | Rainy 2015 | ψ^A^ (shrub cover) ψ^B^ (shrub cover) *p*^A^ (tree cover) *p*^B^ (distance to urban structure) | 852.42 | 0.00 | 0.76 | 8 |
|  |  | ψ^A^ (shrub cover) ψ^BA^ (shrub cover) ψ^Ba^ (shrub cover) *p*^A^ (tree cover) *p*^B^ (distance to urban structure) | 854.67 | 2.26 | 0.24 | 10 |
|  |  |  |  |  |  |  |
|  | Cold-dry 2016 | ψ^A^ (shrub cover) ψ^B^ (shrub cover) *p*^A^ (gardening activities) *p*^B^ (distance to urban structure) | 1013.96 | 0.00 | 0.85 | 8 |
|  |  | ψ^A^ (shrub cover) ψ^BA^ (shrub cover) ψ^Ba^ (shrub cover) *p*^A^ (gardening activities) *p*^B^ (distance to urban structure) | 1017.39 | 3.44 | 0.15 | 10 |
|  |  |  |  |  |  |  |
|  | Warm-dry 2016 | ψ^A^ (shrub cover) ψ^BA^ (shrub cover) ψ^Ba^ (shrub cover) *p*^A^ (shrub cover) *p*^B^ (shrub cover) | 957.66 | 0.00 | 0.90 | 10 |
|  |  | ψ^A^ (shrub cover) ψ^B^ (shrub cover) *p*^A^ (shrub cover) *p*^B^ (shrub cover) | 962.14 | 4.48 | 0.10 | 8 |
|  |  |  |  |  |  |  |
|  | Rainy 2016 | ψ^A^ (shrub cover) ψ^BA^ (shrub cover) ψ^Ba^ (shrub cover) *p*^A^ (exposed rock) *p*^B^ (distance to urban structure) | 1172.36 | 0.00 | 0.55 | 10 |
|  |  | ψ^A^ (shrub cover) ψ^B^ (shrub cover) *p*^A^ (exposed rock) *p*^B^ (distance to urban structure) | 1172.75 | 0.39 | 0.45 | 8 |
|  |  |  |  |  |  |  |
|  | Cold-dry 2017 | ψ^A^ (shrub cover) ψ^B^ (shrub cover) *p*^A^ (tree cover) *p*^B^ (shrub cover) | 1054.06 | 0.00 | 0.55 | 8 |
|  |  | ψ^A^ (shrub cover) ψ^BA^ (shrub cover) ψ^Ba^ (shrub cover) *p*^A^ (tree cover) *p*^B^ (shrub cover) | 1054.47 | 0.41 | 0.45 | 10 |
|  |  |  |  |  |  |  |
| A – Kikuyu grass  (*Pennisetum clandestinum*) | Warm-dry 2015 | ψ^A^ (shrub cover) ψ^B^ (distance to urban structure) *p*^A^ (distance to urban structure) *p*^B^ (shrub cover) | 1211.48 | 0.00 | 0.89 | 8 |
| B – Muhly grass  (*Muhlenbergia robusta*) |  | ψ^A^ (shrub cover) ψ^BA^ (distance to urban structure) ψ^Ba^ (distance to urban structure) *p*^A^ (distance to urban structure) *p*^B^ (shrub cover) | 1215.58 | 4.10 | 0.11 | 10 |
|  |  |  |  |  |  |  |
|  | Rainy 2015 | ψ^A^ (shrub cover) ψ^B^ (shrub cover) *p*^A^ (type of area) *p*^B^ (distance to urban structure) | 814.46 | 0.00 | 0.87 | 9 |
|  |  | ψ^A^ (shrub cover) ψ^BA^ (shrub cover) ψ^Ba^ (shrub cover) *p*^A^ (type of area) *p*^B^ (distance to urban structure) | 818.21 | 3.75 | 0.13 | 11 |
|  |  |  |  |  |  |  |
|  | Cold-dry 2016 | ψ^A^ (shrub cover) ψ^B^ (shrub cover) *p*^A^ (gardening activities) *p*^B^ (distance to urban structure) | 1013.96 | 0.00 | 0.85 | 8 |
|  |  | ψ^A^ (shrub cover) ψ^BA^ (shrub cover) ψ^Ba^ (shrub cover) *p*^A^ (gardening activities) *p*^B^ (distance to urban structure) | 1017.39 | 3.44 | 0.15 | 10 |
|  |  |  |  |  |  |  |
|  | Warm-dry 2016 | ψ^A^ (shrub cover) ψ^B^ (shrub cover) *p*^A^ (herb cover) *p*^B^ (shrub cover) | 1077.22 | 0.00 | 0.78 | 8 |
|  |  | ψ^A^ (shrub cover) ψ^BA^ (shrub cover) ψ^Ba^ (shrub cover) *p*^A^ (herb cover) *p*^B^ (shrub cover) | 1079.79 | 2.57 | 0.22 | 10 |
|  |  |  |  |  |  |  |
|  | Rainy 2016 | ψ^A^ (shrub cover) ψ^B^ (shrub cover) p^A^ (shrub cover) p^B^ (distance to urban structure) | 1395.34 | 0.00 | 0.70 | 8 |
|  |  | ψ^A^ (shrub cover) ψ^BA^ (shrub cover) ψ^Ba^ (shrub cover) *p*^A^ (shrub cover) *p*^B^ (distance to urban structure) | 1397.03 | 1.69 | 0.30 | 10 |
|  |  |  |  |  |  |  |
|  | Cold-dry 2017 | ψ^A^ (gardening activities) ψ^B^ (shrub cover) *p*^A^ (shrub cover) *p*^B^ (shrub cover) | 1261.27 | 0.00 | 0.84 | 8 |
|  |  | ψ^A^ (gardening activities) ψ^BA^ (shrub cover) ψ^Ba^ (shrub cover) *p*^A^ (shrub cover) *p*^B^ (shrub cover) | 1264.61 | 3.34 | 0.16 | 10 |
|  |  |  |  |  |  |  |
| A – Peruvian pepper  (*Schinus molle*) | Warm-dry 2015 | ψ^A^ (tree and shrub richness) ψ^B^ (shrub cover) *p*^A^ (type of area) *p*^B^ (shrub cover) | 1169.84 | 0.00 | 0.84 | 9 |
| B – Tepozan tree  (*Buddleia cordata*) |  | ψ^A^ (tree and shrub richness) ψ^BA^ (shrub cover) ψ^Ba^ (shrub cover) *p*^A^ (type of area) *p*^B^ (shrub cover) | 1173.09 | 3.25 | 0.16 | 11 |
|  |  |  |  |  |  |  |
|  | Rainy 2015 | ψ^A^ (type of area) ψ^B^ (shrub cover) *p*^A^ (exposed rock) *p*^B^ (shrub cover) | 1131.76 | 0.00 | 0.59 | 9 |
|  |  | ψ^A^ (type of area) ψ^BA^ (shrub cover) ψ^Ba^ (shrub cover) *p*^A^ (exposed rock) *p*^B^ (shrub cover) | 1132.51 | 0.75 | 0.41 | 11 |
|  |  |  |  |  |  |  |
|  | Cold-dry 2016 | ψ^A^ (tree and shrub richness) ψ^BA^ (tree and shrub richness) ψ^Ba^ (tree and shrub richness) *p*^A^ (type of area) *p*^B^ (type of area) | 1128.14 | 0.00 | 0.57 | 12 |
|  |  | ψ^A^ (tree and shrub richness) ψ^B^ (tree and shrub richness) *p*^A^ (type of area) *p*^B^ (type of area) | 1128.73 | 0.59 | 0.43 | 10 |
|  |  |  |  |  |  |  |
|  | Warm-dry 2016 | ψ^A^ (tree and shrub richness) ψ^BA^ (tree and shrub richness) ψ^Ba^ (tree and shrub richness) *p*^A^ (gardening activities) *p*^B^ (type of area) | 1120.30 | 0.00 | 0.59 | 11 |
|  |  | ψ^A^ (tree and shrub richness) ψ^B^ (tree and shrub richness) *p*^A^ (gardening activities) *p*^B^ (type of area) | 1121.00 | 0.71 | 0.41 | 9 |
|  |  |  |  |  |  |  |
|  | Rainy 2016 | ψ^A^ (gardening activities) ψ^B^ (tree and shrub richness) *p*^A^ (tree and shrub richness) *p*^B^ (shrub cover) | 1515.54 | 0.00 | 0.73 | 8 |
|  |  | ψ^A^ (gardening activities) ψ^BA^ (tree and shrub richness) ψ^Ba^ (tree and shrub richness) *p*^A^ (tree and shrub richness) *p*^B^ (shrub cover) | 1517.54 | 2.00 | 0.27 | 10 |
|  |  |  |  |  |  |  |
|  | Cold-dry 2017 | ψ^A^ (tree and shrub richness) ψ^B^ (shrub cover) *p*^A^ (gardening activities) *p*^B^ (type of area) | 1369.38 | 0.00 | 0.79 | 9 |
|  |  | ψ^A^ (tree and shrub richness) ψ^BA^ (shrub cover) ψ^Ba^ (shrub cover) *p*^A^ (gardening activities) *p*^B^ (type of area) | 1372.06 | 2.67 | 0.21 | 11 |
|  |  |  |  |  |  |  |
| A – Peruvian pepper  (*Schinus molle*) | Warm-dry 2015 | ψ^A^ (tree and shrub richness) ψ^B^ (shrub cover) *p*^A^ (type of area) *p*^B^ (type of area) *r*^B^ (type of area) | 1303.93 | 0.00 | 0.86 | 13 |
| B – House finch  (*Haemorhous mexicanus*) |  | ψ^A^ (tree and shrub richness) ψ^BA^ (shrub cover) ψ^Ba^ (shrub cover) *p*^A^ (type of area) *p*^B^ (type of area) *r*^B^ (type of area) | 1307.63 | 3.70 | 0.14 | 15 |
|  |  |  |  |  |  |  |
|  | Rainy 2015 | ψ^A^ (type of area) ψ^BA^ (type of area) ψ^Ba^ (type of area) *p*^A^ (exposed rock) *p*^B^ (herb cover) *r*^B^ (herb cover) | 1181.87 | 0.00 | 0.62 | 15 |
|  |  | ψ^A^ (type of area) ψ^B^ (type of area) *p*^A^ (exposed rock) *p*^B^ (herb cover) *r*^B^ (herb cover) | 1182.86 | 0.99 | 0.38 | 12 |
|  |  |  |  |  |  |  |
|  | Cold-dry 2016 | ψ^A^ (tree and shrub richness) ψ^B^ (shrub cover) *p*^A^ (type of area) *p*^B^ (shrub cover) *r*^B^ (shrub cover) | 1172.16 | 0.00 | 0.93 | 11 |
|  |  | ψ^A^ (tree and shrub richness) ψ^BA^ (shrub cover) ψ^Ba^ (shrub cover) *p*^A^ (type of area) *p*^B^ (shrub cover) *r*^B^ (shrub cover) | 1177.28 | 5.12 | 0.07 | 13 |
|  |  |  |  |  |  |  |
|  | Warm-dry 2016 | ψ^A^ (tree and shrub richness) ψ^B^ (intercept only) *p*^A^ (gardening activities) *p*^B^ (shrub cover) *r*^B^ (shrub cover) | 1311.24 | 0.00 | 0.77 | 9 |
|  |  | ψ^A^ (tree and shrub richness) ψ^BA^ (intercept only) ψ^Ba^ (intercept only) *p*^A^ (gardening activities) *p*^B^ (shrub cover) *r*^B^ (shrub cover) | 1313.62 | 2.38 | 0.23 | 10 |
|  |  |  |  |  |  |  |
|  | Rainy 2016 | ψ^A^ (gardening activities) ψ^B^ (intercept only) *p*^A^ (tree and shrub richness) *p*^B^ (distance to urban structure) *r*^B^ (distance to urban structure) | 1774.71 | 0.00 | 0.77 | 9 |
|  |  | ψ^A^ (gardening activities) ψ^BA^ (intercept only) ψ^Ba^ (intercept only) *p*^A^ (tree and shrub richness) *p*^B^ (distance to urban structure) *r*^B^ (distance to urban structure) | 1777.18 | 2.47 | 0.23 | 10 |
|  |  |  |  |  |  |  |
|  | Cold-dry 2017 | ψ^A^ (tree and shrub richness) ψ^B^ (type of area) *p*^A^ (gardening activities) *p*^B^ (distance to urban structure) *r*^B^ (distance to urban structure) | 1418.61 | 0.00 | 0.88 | 11 |
|  |  | ψ^A^ (tree and shrub richness) ψ^BA^ (type of area) ψ^Ba^ (intercept only) *p*^A^ (gardening activities) *p*^B^ (distance to urban structure) *r*^B^ (distance to urban structure) | 1422.57 | 3.95 | 0.12 | 14 |
|  |  |  |  |  |  |  |
| A – Peruvian pepper  (*Schinus molle*) | Warm-dry 2015 | ψ^A^ (tree and shrub richness) ψ^B^ (distance to urban structure) *p*^A^ (type of area) *p*^B^ (tree cover) *r*^B^ (tree cover) | 912.99 | 0.00 | 0.91 | 11 |
| B – Grey silky-flycatcher (*Ptiliogonys cinereus*) |  | ψ^A^ (tree and shrub richness) ψ^BA^ (distance to urban structure) ψ^Ba^ (distance to urban structure) *p*^A^ (type of area) *p*^B^ (tree cover) *r*^B^ (tree cover) | 917.69 | 4.70 | 0.09 | 13 |
|  |  |  |  |  |  |  |
|  | Rainy 2015 | ψ^A^ (type of area) ψ^B^ (shrub cover) *p*^A^ (exposed rock) *p*^B^ (distance to urban structure) *r*^B^ (distance to urban structure) | 873.17 | 0.00 | 0.90 | 11 |
|  |  | ψ^A^ (type of area) ψ^BA^ (shrub cover) ψ^Ba^ (shrub cover) *p*^A^ (exposed rock) *p*^B^ (distance to urban structure) *r*^B^ (distance to urban structure) | 877.57 | 4.40 | 0.10 | 13 |
|  |  |  |  |  |  |  |
|  | Cold-dry 2016 | ψ^A^ (tree and shrub richness) ψ^B^ (intercept only) *p*^A^ (type of area) *p*^B^ (shrub cover) *r*^B^ (shrub cover) | 856.28 | 0.00 | 0.77 | 10 |
|  |  | ψ^A^ (tree and shrub richness) ψ^BA^ (intercept only) ψ^Ba^ (intercept only) *p*^A^ (type of area) *p*^B^ (shrub cover) *r*^B^ (shrub cover) | 858.70 | 2.43 | 0.23 | 11 |
|  |  |  |  |  |  |  |
|  | Warm-dry 2016 | ψ^A^ (tree and shrub richness) ψ^B^ (distance to urban structure) *p*^A^ (gardening activities) *p*^B^ (herb cover) *r*^B^ (herb cover) | 1067.66 | 0.00 | 0.86 | 10 |
|  |  | ψ^A^ (tree and shrub richness) ψ^BA^ (distance to urban structure) ψ^Ba^ (distance to urban structure) *p*^A^ (gardening activities) *p*^B^ (herb cover) *r*^B^ (herb cover) | 1071.25 | 3.60 | 0.14 | 12 |
|  |  |  |  |  |  |  |
|  | Rainy 2016 | ψ^A^ (gardening activities) ψ^B^ (gardening activities) *p*^A^ (tree and shrub richness) *p*^B^ (distance to urban structure) *r*^B^ (distance to urban structure) | 1208.11 | 0.00 | 0.57 | 10 |
|  |  | ψ^A^ (gardening activities) ψ^BA^ (gardening activities) ψ^Ba^ (gardening activities) *p*^A^ (tree and shrub richness) *p*^B^ (distance to urban structure) *r*^B^ (distance to urban structure) | 1208.66 | 0.55 | 0.43 | 12 |
|  |  |  |  |  |  |  |
|  | Cold-dry 2017 | ψ^A^ (tree and shrub richness) ψ^B^ (distance to urban structure) *p*^A^ (gardening activities) *p*^B^ (tree richness) *r*^B^ (tree richness) | 1143.57 | 0.00 | 0.72 | 10 |
|  |  | ψ^A^ (tree and shrub richness) ψ^BA^ (distance to urban structure) ψ^Ba^ (distance to urban structure) *p*^A^ (gardening activities) *p*^B^ (tree richness) *r*^B^ (tree richness) | 1145.44 | 1.87 | 0.28 | 12 |
|  |  |  |  |  |  |  |
| A – Peruvian pepper  (*Schinus molle*) | Warm-dry 2015 | ψ^A^ (tree and shrub richness) ψ^B^ (distance to urban structure) *p*^A^ (type of area) *p*^B^ (distance to urban structure) *r*^B^ (distance to urban structure) | 1076.58 | 0.00 | 0.72 | 11 |
| B – Bushtit  (*Psaltriparus minimus*) |  | ψ^A^ (tree and shrub richness) ψ^BA^ (distance to urban structure) ψ^Ba^ (distance to urban structure) *p*^A^ (type of area) *p*^B^ (distance to urban structure) *r*^B^ (distance to urban structure) | 1078.48 | 1.90 | 0.28 | 13 |
|  |  |  |  |  |  |  |
|  | Rainy 2015 | ψ^A^ (type of area) ψ^B^ (tree and shrub richness) *p*^A^ (exposed rock) *p*^B^ (distance to urban structure) *r*^B^ (distance to urban structure) | 945.25 | 0.00 | 0.87 | 11 |
|  |  | ψ^A^ (type of area) ψ^BA^ (tree and shrub richness) ψ^Ba^ (tree and shrub richness) *p*^A^ (exposed rock) *p*^B^ (distance to urban structure) *r*^B^ (distance to urban structure) | 949.05 | 3.80 | 0.13 | 13 |
|  |  |  |  |  |  |  |
|  | Cold-dry 2016 | ψ^A^ (tree and shrub richness) ψ^B^ (intercept only) *p*^A^ (type of area) *p*^B^ (gardening activities) *r*^B^ (gardening activities) | 766.65 | 0.00 | 0.76 | 10 |
|  |  | ψ^A^ (tree and shrub richness) ψ^BA^ (intercept only) ψ^Ba^ (intercept only) *p*^A^ (type of area) *p*^B^ (gardening activities) *r*^B^ (gardening activities) | 768.90 | 2.26 | 0.24 | 11 |
|  |  |  |  |  |  |  |
|  | Warm-dry 2016 | ψ^A^ (tree and shrub richness) ψ^B^ (distance to urban structure) *p*^A^ (gardening activities) *p*^B^ (shrub cover) *r*^B^ (shrub cover) | 947.44 | 0.00 | 0.86 | 10 |
|  |  | ψ^A^ (tree and shrub richness) ψ^BA^ (distance to urban structure) ψ^Ba^ (distance to urban structure) *p*^A^ (gardening activities) *p*^B^ (shrub cover) *r*^B^ (shrub cover) | 951.07 | 3.63 | 0.14 | 12 |
|  |  |  |  |  |  |  |
|  | Rainy 2016 | ψ^A^ (gardening activities) ψ^BA^ (gardening activities) ψ^Ba^ (gardening activities) *p*^A^ (tree and shrub richness) *p*^B^ (intercept only) *r*^B^ (intercept only) | 1281.88 | 0.00 | 0.57 | 10 |
|  |  | ψ^A^ (gardening activities) ψ^B^ (gardening activities) *p*^A^ (tree and shrub richness) *p*^B^ (intercept only) *r*^B^ (intercept only) | 1282.47 | 0.60 | 0.43 | 8 |
|  |  |  |  |  |  |  |
|  | Cold-dry 2017 | ψ^A^ (tree and shrub richness) ψ^B^ (distance to urban structure) *p*^A^ (gardening activities) *p*^B^ (distance to urban structure) *r*^B^ (distance to urban structure) | 1019.39 | 0.00 | 0.92 | 10 |
|  |  | ψ^A^ (tree and shrub richness) ψ^BA^ (distance to urban structure) ψ^Ba^ (distance to urban structure) *p*^A^ (gardening activities) *p*^B^ (distance to urban structure) *r*^B^ (distance to urban structure) | 1024.24 | 4.86 | 0.08 | 12 |
|  |  |  |  |  |  |  |
| A – River red gum  (*Eucalyptus camaldulensis*) | Warm-dry 2015 | ψ^A^ (distance to urban structure) ψ^B^ (shrub cover) *p*^A^ (herb cover) *p*^B^ (type of area) *r*^B^ (type of area) | 1161.37 | 0.00 | 0.72 | 12 |
| B – House finch  (*Haemorhous mexicanus*) |  | ψ^A^ (distance to urban structure) ψ^BA^ (shrub cover) ψ^Ba^ (shrub cover) *p*^A^ (herb cover) *p*^B^ (type of area) *r*^B^ (type of area) | 1163.27 | 1.90 | 0.28 | 14 |
|  |  |  |  |  |  |  |
|  | Rainy 2015 | ψ^A^ (distance to urban structure) ψ^B^ (type of area) *p*^A^ (tree and shrub richness) *p*^B^ (herb cover) *r*^B^ (herb cover) | 945.73 | 0.00 | 0.92 | 11 |
|  |  | ψ^A^ (distance to urban structure) ψ^BA^ (type of area) ψ^Ba^ (type of area) *p*^A^ (tree and shrub richness) *p*^B^ (herb cover) *r*^B^ (herb cover) | 950.50 | 4.77 | 0.08 | 14 |
|  |  |  |  |  |  |  |
|  | Cold-dry 2016 | ψ^A^ (distance to urban structure) ψ^BA^ (shrub cover) ψ^Ba^ (shrub cover) *p*^A^ (tree richness) *p*^B^ (shrub cover) *r*^B^ (shrub cover) | 1013.72 | 0.00 | 0.60 | 12 |
|  |  | ψ^A^ (distance to urban structure) ψ^B^ (shrub cover) *p*^A^ (tree richness) *p*^B^ (shrub cover) *r*^B^ (shrub cover) | 1014.54 | 0.83 | 0.40 | 10 |
|  |  |  |  |  |  |  |
|  | Warm-dry 2016 | ψ^A^ (distance to urban structure) ψ^B^ (intercept only) *p*^A^ (type of area) *p*^B^ (shrub cover) *r*^B^ (shrub cover) | 1267.02 | 0.00 | 0.73 | 10 |
|  |  | ψ^A^ (distance to urban structure) ψ^BA^ (intercept only) ψ^Ba^ (intercept only) *p*^A^ (type of area) *p*^B^ (shrub cover) *r*^B^ (shrub cover) | 1268.99 | 1.97 | 0.27 | 11 |
|  |  |  |  |  |  |  |
|  | Rainy 2016 | ψ^A^ (distance to urban structure) ψ^B^ (intercept only) *p*^A^ (tree richness) *p*^B^ (distance to urban structure) *r*^B^ (distance to urban structure) | 1628.08 | 0.00 | 0.74 | 9 |
|  |  | ψ^A^ (distance to urban structure) ψ^BA^ (intercept only) ψ^Ba^ (intercept only) *p*^A^ (tree richness) *p*^B^ (distance to urban structure) *r*^B^ (distance to urban structure) | 1630.13 | 2.04 | 0.26 | 10 |
|  |  |  |  |  |  |  |
|  | Cold-dry 2017 | ψ^A^ (distance to urban structure) ψ^B^ (type of area) *p*^A^ (type of area) *p*^B^ (distance to urban structure) *r*^B^ (distance to urban structure) | 1318.54 | 0.00 | 0.96 | 12 |
|  |  | ψ^A^ (distance to urban structure) ψ^BA^ (type of area) ψ^Ba^ (type of area) *p*^A^ (type of area) *p*^B^ (distance to urban structure) *r*^B^ (distance to urban structure) | 1324.81 | 6.27 | 0.04 | 15 |
|  |  |  |  |  |  |  |
| A – River red gum  (*Eucalyptus camaldulensis*) | Warm-dry 2015 | ψ^A^ (distance to urban structure) ψ^BA^ (distance to urban structure) ψ^Ba^ (distance to urban structure) *p*^A^ (herb cover) *p*^B^ (tree cover) *r*^B^ (tree cover) | 774.18 | 0.00 | 0.87 | 12 |
| B – Grey silky-flycatcher  (*Ptiliogonys cinereus*) |  | ψ^A^ (distance to urban structure) ψ^B^ (distance to urban structure) *p*^A^ (herb cover) *p*^B^ (tree cover) *r*^B^ (tree cover) | 778.01 | 3.83 | 0.13 | 10 |
|  |  |  |  |  |  |  |
|  | Rainy 2015 | ψ^A^ (distance to urban structure) ψ^B^ (shrub cover) *p*^A^ (tree and shrub richness) *p*^B^ (distance to urban structure) *r*^B^ (distance to urban structure) | 628.00 | 0.00 | 0.81 | 10 |
|  |  | ψ^A^ (distance to urban structure) ψ^BA^ (shrub cover) ψ^Ba^ (shrub cover) *p*^A^ (tree and shrub richness) *p*^B^ (distance to urban structure) *r*^B^ (distance to urban structure) | 630.94 | 2.93 | 0.19 | 12 |
|  |  |  |  |  |  |  |
|  | Cold-dry 2016 | ψ^A^ (distance to urban structure) ψ^BA^ (intercept only) ψ^Ba^ (intercept only) *p*^A^ (tree richness) *p*^B^ (shrub cover) *r*^B^ (shrub cover) | 700.58 | 0.00 | 0.73 | 10 |
|  |  | ψ^A^ (distance to urban structure) ψ^B^ (intercept only) *p*^A^ (tree richness) *p*^B^ (shrub cover) *r*^B^ (shrub cover) | 702.60 | 2.02 | 0.27 | 9 |
|  |  |  |  |  |  |  |
|  | Warm-dry 2016 | ψ^A^ (distance to urban structure) ψ^B^ (distance to urban structure) *p*^A^ (type of area) *p*^B^ (herb cover) *r*^B^ (herb cover) | 1012.83 | 0.00 | 0.93 | 11 |
|  |  | ψ^A^ (distance to urban structure) ψ^BA^ (distance to urban structure) ψ^Ba^ (distance to urban structure) *p*^A^ (type of area) *p*^B^ (herb cover) *r*^B^ (herb cover) | 1018.01 | 5.18 | 0.07 | 13 |
|  |  |  |  |  |  |  |
|  | Rainy 2016 | ψ^A^ (distance to urban structure) ψ^B^ (gardening activities) *p*^A^ (tree richness) *p*^B^ (distance to urban structure) *r*^B^ (distance to urban structure) | 1049.37 | 0.00 | 0.87 | 10 |
|  |  | ψ^A^ (distance to urban structure) ψ^BA^ (gardening activities) ψ^Ba^ (gardening activities) *p*^A^ (tree richness) *p*^B^ (distance to urban structure) *r*^B^ (distance to urban structure) | 1053.25 | 3.88 | 0.13 | 12 |
|  |  |  |  |  |  |  |
|  | Cold-dry 2017 | ψ^A^ (distance to urban structure) ψ^B^ (distance to urban structure) *p*^A^ (type of area) *p*^B^ (tree richness) *r*^B^ (tree richness) | 1049.91 | 0.00 | 0.93 | 11 |
|  |  | ψ^A^ (distance to urban structure) ψ^BA^ (distance to urban structure) ψ^Ba^ (distance to urban structure) *p*^A^ (type of area) *p*^B^ (tree richness) *r*^B^ (tree richness) | 1055.04 | 5.13 | 0.07 | 13 |
|  |  |  |  |  |  |  |
| A – River red gum  (*Eucalyptus camaldulensis*) | Warm-dry 2015 | ψ^A^ (distance to urban structure) ψ^B^ (distance to urban structure) *p*^A^ (herb cover) *p*^B^ (distance to urban structure) *r*^B^ (distance to urban structure) | 926.99 | 0.00 | 0.88 | 10 |
| B – Bushtit  (*Psaltriparus minimus*) |  | ψ^A^ (distance to urban structure) ψ^BA^ (distance to urban structure) ψ^Ba^ (distance to urban structure) *p*^A^ (herb cover) *p*^B^ (distance to urban structure) *r*^B^ (distance to urban structure) | 931.06 | 4.07 | 0.12 | 12 |
|  |  |  |  |  |  |  |
|  | Rainy 2015 | ψ^A^ (distance to urban structure) ψ^B^ (tree and shrub richness) *p*^A^ (tree and shrub richness) *p*^B^ (distance to urban structure) *r*^B^ (distance to urban structure) | 711.23 | 0.00 | 0.90 | 10 |
|  |  | ψ^A^ (distance to urban structure) ψ^BA^ (tree and shrub richness) ψ^Ba^ (tree and shrub richness) *p*^A^ (tree and shrub richness) *p*^B^ (distance to urban structure) *r*^B^ (distance to urban structure) | 715.70 | 4.47 | 0.10 | 12 |
|  |  |  |  |  |  |  |
|  | Cold-dry 2016 | ψ^A^ (distance to urban structure) ψ^B^ (intercept only) *p*^A^ (tree richness) *p*^B^ (gardening activities) *r*^B^ (gardening activities) | 609.51 | 0.00 | 0.76 | 9 |
|  |  | ψ^A^ (distance to urban structure) ψ^BA^ (intercept only) ψ^Ba^ (intercept only) *p*^A^ (tree richness) *p*^B^ (gardening activities) *r*^B^ (gardening activities) | 611.86 | 2.35 | 0.24 | 10 |
|  |  |  |  |  |  |  |
|  | Warm-dry 2016 | ψ^A^ (distance to urban structure) ψ^B^ (distance to urban structure) *p*^A^ (type of area) *p*^B^ (shrub cover) *r*^B^ (shrub cover) | 908.21 | 0.00 | 0.91 | 11 |
|  |  | ψ^A^ (distance to urban structure) ψ^BA^ (distance to urban structure) ψ^Ba^ (distance to urban structure) *p*^A^ (type of area) *p*^B^ (shrub cover) *r*^B^ (shrub cover) | 912.78 | 4.57 | 0.09 | 13 |
|  |  |  |  |  |  |  |
|  | Rainy 2016 | ψ^A^ (distance to urban structure) ψ^B^ (gardening activities) *p*^A^ (tree richness) *p*^B^ (intercept only) *r*^B^ (intercept only) | 1127.31 | 0.00 | 0.88 | 8 |
|  |  | ψ^A^ (distance to urban structure) ψ^BA^ (gardening activities) ψ^Ba^ (gardening activities) *p*^A^ (tree richness) *p*^B^ (intercept only) *r*^B^ (intercept only) | 1131.38 | 4.07 | 0.12 | 10 |
|  |  |  |  |  |  |  |
|  | Cold-dry 2017 | ψ^A^ (distance to urban structure) ψ^B^ (distance to urban structure) *p*^A^ (type of area) *p*^B^ (distance to urban structure) *r*^B^ (distance to urban structure) | 920.84 | 0.00 | 0.90 | 11 |
|  |  | ψ^A^ (distance to urban structure) ψ^BA^ (distance to urban structure) ψ^Ba^ (distance to urban structure) *p*^A^ (type of area) *p*^B^ (distance to urban structure) *r*^B^ (distance to urban structure) | 925.34 | 4.50 | 0.10 | 13 |
|  |  |  |  |  |  |  |
| A – Tropical ash  (*Fraxinus uhdei*) | Warm-dry 2015 | ψ^A^ (shrub cover) ψ^B^ (shrub cover) *p*^A^ (gardening activities) *p*^B^ (type of area) *r*^B^ (type of area) | 1170.02 | 0.00 | 0.89 | 12 |
| B – House finch  (*Haemorhous mexicanus*) |  | ψ^A^ (shrub cover) ψ^BA^ (shrub cover) ψ^Ba^ (shrub cover) *p*^A^ (gardening activities) *p*^B^ (type of area) *r*^B^ (type of area) | 1174.16 | 4.14 | 0.11 | 14 |
|  |  |  |  |  |  |  |
|  | Rainy 2015 | ψ^A^ (exposed rock) ψ^B^ (type of area) *p*^A^ (type of area) *p*^B^ (herb cover) *r*^B^ (herb cover) | 1007.04 | 0.00 | 0.96 | 12 |
|  |  | ψ^A^ (exposed rock) ψ^BA^ (type of area) ψ^Ba^ (type of area) *p*^A^ (type of area) *p*^B^ (herb cover) *r*^B^ (herb cover) | 1013.67 | 6.63 | 0.04 | 15 |
|  |  |  |  |  |  |  |
|  | Cold-dry 2016 | ψ^A^ (type of area) ψ^BA^ (shrub cover) ψ^Ba^ (shrub cover) *p*^A^ (shrub cover) *p*^B^ (shrub cover) *r*^B^ (shrub cover) | 1002.10 | 0.00 | 0.98 | 13 |
|  |  | ψ^A^ (type of area) ψ^B^ (shrub cover) *p*^A^ (shrub cover) *p*^B^ (shrub cover) *r*^B^ (shrub cover) | 1009.72 | 7.62 | 0.02 | 11 |
|  |  |  |  |  |  |  |
|  | Warm-dry 2016 | ψ^A^ (tree richness) ψ^BA^ (intercept only) ψ^Ba^ (intercept only) *p*^A^ (type of area) *p*^B^ (distance to urban structure) *r*^B^ (distance to urban structure) | 1319.41 | 0.00 | 0.63 | 11 |
|  |  | ψ^A^ (tree richness) ψ^B^ (intercept only) *p*^A^ (type of area) *p*^B^ (distance to urban structure) *r*^B^ (distance to urban structure) | 1320.50 | 1.09 | 0.37 | 10 |
|  |  |  |  |  |  |  |
|  | Rainy 2016 | ψ^A^ (type of area) ψ^BA^ (intercept only) ψ^Ba^ (intercept only) *p*^A^ (shrub cover) *p*^B^ (distance to urban structure) *r*^B^ (distance to urban structure) | 1639.51 | 0.00 | 0.63 | 11 |
|  |  | ψ^A^ (type of area) ψ^B^ (intercept only) *p*^A^ (shrub cover) *p*^B^ (distance to urban structure) *r*^B^ (distance to urban structure) | 1640.56 | 1.06 | 0.37 | 10 |
|  |  |  |  |  |  |  |
|  | Cold-dry 2017 | ψ^A^ (gardening activities) ψ^B^ (type of area) *p*^A^ (shrub cover) *p*^B^ (distance to urban structure) *r*^B^ (distance to urban structure) | 1420.38 | 0.00 | 0.98 | 11 |
|  |  | ψ^A^ (gardening activities) ψ^BA^ (type of area) ψ^Ba^ (type of area) *p*^A^ (shrub cover) *p*^B^ (distance to urban structure) *r*^B^ (distance to urban structure) | 1427.81 | 7.43 | 0.02 | 14 |
|  |  |  |  |  |  |  |
| A – Tropical ash  (*Fraxinus uhdei*) | Warm-dry 2015 | ψ^A^ (shrub cover) ψ^B^ (distance to urban structure) *p*^A^ (gardening activities) *p*^B^ (tree cover) *r*^B^ (tree cover) | 786.09 | 0.00 | 0.91 | 10 |
| B – Grey silky-flycatcher  (*Ptiliogonys cinereus*) |  | ψ^A^ (shrub cover) ψ^BA^ (distance to urban structure) ψ^Ba^ (distance to urban structure) *p*^A^ (gardening activities) *p*^B^ (tree cover) *r*^B^ (tree cover) | 790.60 | 4.51 | 0.09 | 12 |
|  |  |  |  |  |  |  |
|  | Rainy 2015 | ψ^A^ (exposed rock) ψ^B^ (shrub cover) *p*^A^ (type of area) *p*^B^ (distance to urban structure) *r*^B^ (distance to urban structure) | 694.27 | 0.00 | 0.92 | 11 |
|  |  | ψ^A^ (exposed rock) ψ^BA^ (shrub cover) ψ^Ba^ (shrub cover) *p*^A^ (type of area) *p*^B^ (distance to urban structure) *r*^B^ (distance to urban structure) | 699.13 | 4.86 | 0.08 | 13 |
|  |  |  |  |  |  |  |
|  | Cold-dry 2016 | ψ^A^ (type of area) ψ^B^ (intercept only) *p*^A^ (shrub cover) *p*^B^ (shrub cover) *r*^B^ (shrub cover) | 698.44 | 0.00 | 0.69 | 10 |
|  |  | ψ^A^ (type of area) ψ^BA^ (intercept only) ψ^Ba^ (intercept only) *p*^A^ (shrub cover) *p*^B^ (shrub cover) *r*^B^ (shrub cover) | 700.05 | 1.61 | 0.31 | 11 |
|  |  |  |  |  |  |  |
|  | Warm-dry 2016 | ψ^A^ (tree richness) ψ^B^ (distance to urban structure) *p*^A^ (type of area) *p*^B^ (herb cover) *r*^B^ (herb cover) | 1085.87 | 0.00 | 0.93 | 11 |
|  |  | ψ^A^ (tree richness) ψ^BA^ (distance to urban structure) ψ^Ba^ (distance to urban structure) *p*^A^ (type of area) *p*^B^ (herb cover) *r*^B^ (herb cover) | 1090.91 | 5.04 | 0.07 | 13 |
|  |  |  |  |  |  |  |
|  | Rainy 2016 | ψ^A^ (type of area) ψ^B^ (gardening activities) *p*^A^ (shrub cover) *p*^B^ (distance to urban structure) *r*^B^ (distance to urban structure) | 1063.80 | 0.00 | 0.82 | 11 |
|  |  | ψ^A^ (type of area) ψ^BA^ (gardening activities) ψ^Ba^ (gardening activities) *p*^A^ (shrub cover) *p*^B^ (distance to urban structure) *r*^B^ (distance to urban structure) | 1066.79 | 2.99 | 0.18 | 13 |
|  |  |  |  |  |  |  |
|  | Cold-dry 2017 | ψ^A^ (gardening activities) ψ^BA^ (distance to urban structure) ψ^Ba^ (distance to urban structure) *p*^A^ (shrub cover) *p*^B^ (tree richness) *r*^B^ (tree richness) | 1149.36 | 0.00 | 0.95 | 12 |
|  |  | ψ^A^ (gardening activities) ψ^B^ (distance to urban structure) *p*^A^ (shrub cover) *p*^B^ (tree richness) *r*^B^ (tree richness) | 1155.07 | 5.71 | 0.05 | 10 |
|  |  |  |  |  |  |  |
| A – Tropical ash  (*Fraxinus uhdei*) | Warm-dry 2015 | ψ^A^ (shrub cover) ψ^B^ (distance to urban structure) p^A^ (gardening activities) p^B^ (distance to urban structure) r^B^ (distance to urban structure) | 938.75 | 0.00 | 0.88 | 10 |
| B – Bushtit  (*Psaltriparus minimus*) |  | ψ^A^ (shrub cover) ψ^BA^ (distance to urban structure) ψ^Ba^ (shrub cover) p^A^ (gardening activities) p^B^ (distance to urban structure) r^B^ (distance to urban structure) | 942.66 | 3.91 | 0.12 | 12 |
|  |  |  |  |  |  |  |
|  | Rainy 2015 | ψ^A^ (exposed rock) ψ^B^ (tree and shrub richness) *p*^A^ (type of area) *p*^B^ (distance to urban structure) *r*^B^ (distance to urban structure) | 771.37 | 0.00 | 0.91 | 11 |
|  |  | ψ^A^ (exposed rock) ψ^BA^ (tree and shrub richness) ψ^Ba^ (tree and shrub richness) *p*^A^ (type of area) *p*^B^ (distance to urban structure) *r*^B^ (distance to urban structure) | 776.10 | 4.73 | 0.09 | 13 |
|  |  |  |  |  |  |  |
|  | Cold-dry 2016 | ψ^A^ (type of area) ψ^B^ (intercept only) *p*^A^ (shrub cover) *p*^B^ (gardening activities) *r*^B^ (gardening activities) | 606.51 | 0.00 | 0.72 | 10 |
|  |  | ψ^A^ (type of area) ψ^BA^ (intercept only) ψ^Ba^ (intercept only) *p*^A^ (shrub cover) *p*^B^ (gardening activities) *r*^B^ (gardening activities) | 608.37 | 1.86 | 0.28 | 11 |
|  |  |  |  |  |  |  |
|  | Warm-dry 2016 | ψ^A^ (tree richness) ψ^BA^ (distance to urban structure) ψ^Ba^ (distance to urban structure) *p*^A^ (type of area) *p*^B^ (shrub cover) *r*^B^ (shrub cover) | 963.11 | 0.00 | 0.56 | 13 |
|  |  | ψ^A^ (tree richness) ψ^B^ (distance to urban structure) *p*^A^ (type of area) *p*^B^ (shrub cover) *r*^B^ (shrub cover) | 963.60 | 0.50 | 0.44 | 11 |
|  |  |  |  |  |  |  |
|  | Rainy 2016 | ψ^A^ (type of area) ψ^B^ (gardening activities) *p*^A^ (shrub cover) *p*^B^ (intercept only) *r*^B^ (intercept only) | 1139.45 | 0.00 | 0.91 | 9 |
|  |  | ψ^A^ (type of area) ψ^BA^ (gardening activities) ψ^Ba^ (gardening activities) *p*^A^ (shrub cover) *p*^B^ (intercept only) *r*^B^ (intercept only) | 1144.02 | 4.57 | 0.09 | 11 |
|  |  |  |  |  |  |  |
|  | Cold-dry 2017 | ψ^A^ (gardening activities) ψ^B^ (distance to urban structure) *p*^A^ (shrub cover) *p*^B^ (distance to urban structure) *r*^B^ (distance to urban structure) | 1023.77 | 0.00 | 0.88 | 10 |
|  |  | ψ^A^ (gardening activities) ψ^BA^ (distance to urban structure) ψ^Ba^ (distance to urban structure) *p*^A^ (shrub cover) *p*^B^ (distance to urban structure) *r*^B^ (distance to urban structure) | 1027.67 | 3.90 | 0.12 | 12 |

For each pair of exotic-native species and for each season we used the Akaike’s information criterion adjusted for small sample sizes (AICc) to compare a model in which the occupancy (ψ) of the native species depends on the presence (ψ^BA^) or absence (ψ^Ba^) of the exotic species against a model in which the occupancy of the native species is independent of the presence of the exotic species (ψ^B^). The environmental covariates with strongest effect on the parameters are shown within parentheses.

ψ^A^ = occupancy of the exotic species. *p*^A^ = detection probability of the exotic species. *p*^B^ = detection probability of the native species. *r*^B^ = detection probability of the native species given that the exotic species was also present. *w* = relative support for each model in the data (Akaike weights). *k* = number of parameters.
